# Atacama Large Aperture Submillimeter Telescope (AtLAST) science: Resolving the hot and ionized Universe through the Sunyaev-Zeldovich effect

**DOI:** 10.12688/openreseurope.17449.1

**Published:** 2024-06-10

**Authors:** Luca Di Mascolo, Yvette Perrott, Tony Mroczkowski, Stefano Andreon, Stefano Ettori, Aurora Simionescu, Srinivasan Raghunathan, Joshiwa van Marrewijk, Claudia Cicone, Minju Lee, Dylan Nelson, Laura Sommovigo, Mark Booth, Pamela Klaassen, Paola Andreani, Martin A. Cordiner, Doug Johnstone, Eelco van Kampen, Daizhong Liu, Thomas J. Maccarone, Thomas W. Morris, Amélie Saintonge, Matthew Smith, Alexander E. Thelen, Sven Wedemeyer

**Affiliations:** 1 Laboratoire Lagrange, Observatoire de la Côte d'Azur, University of Côte d'Azur, Nice, Provence-Alpes-Côte d'Azur, 06304, France; 2 Astronomy Unit, Department of Physics, University of Trieste, Trieste, Friuli-Venezia Giulia, 34131, Italy; 3 INAF – Osservatorio Astronomico di Trieste, Trieste, 34014, Italy; 4 IFPU – Institute for Fundamental Physics of the Universe, Trieste, 34014, Italy; 5 Victoria University of Wellington, Wellington, New Zealand; 6 European Southern Observatory, Garching, 85748, Germany; 7 INAF – Osservatorio Astronomico di Brera, Milano, 20121, Italy; 8 INAF – Osservatorio di Astrofisica e Scienza dello Spazio, Bologna, 40129, Italy; 9 INFN – Sezione di Bologna, Bologna, 40127, Italy; 10 SRON Netherlands Institute for Space Research, Niels Bohrweg 4, Leiden, 2333 CA, The Netherlands; 11 Leiden Observatory, Niels Bohrweg 2, Leiden University, Leiden, 2333 CA, The Netherlands; 12 Kavli Institute for the Physics and Mathematics of the Universe, University of Tokyo, Kashiwa, Chiba, 277-8583, Japan; 13 Center for AstroPhysical Surveys, National Center for Supercomputing Applications, Urbana, Illinois, 61801, USA; 14 Institute of Theoretical Astrophysics, University of Oslo, Oslo, 0315, Norway; 15 Cosmic Dawn Center, Copenhagen, Denmark; 16 DTU-Space, Technical University of Denmark, Kongens Lyngby, 2800, Denmark; 17 Zentrum für Astronomie, Institut für Theoretische Astrophysik, Heidelberg University, Heidelberg, Baden-Württemberg, 69120, Germany; 18 Center for Computational Astrophysics, Flatiron Institute, New York, New York, 10010, USA; 19 Scuola Normale Superiore, Pisa, Tuscany, 56126, Italy; 20 UK Astronomy Technology Centre, Royal Observatory Edinburgh, Edinburgh, EH9 3HJ, UK; 21 Astrochemistry Laboratory, Code 691, NASA Goddard Space Flight Center, Greenbelt, Maryland, 20771, USA; 22 NRC Herzberg Astronomy and Astrophysics, Victoria, British Columbia, V9E 2E7, Canada; 23 Department of Physics and Astronomy, University of Victoria, Victoria, British Columbia, V8P 5C2, Canada; 24 Max-Planck-Institut für extraterrestrische Physik, Garching, Bayern, 85748, Germany; 25 Purple Mountain Observatory, Chinese Academy of Sciences, Nanjing, 210023, China; 26 Department of Physics & Astronomy, Texas Tech University, Lubbock, Texas, 79409-1051, USA; 27 Brookhaven National Laboratory, Upton, New York, 11973, USA; 28 Lawrence Berkeley National Laboratory, Berkeley, California, 94720, USA; 29 Department of Physics and Astronomy, University College London, London, England, WC1E 6BT, UK; 30 Max-Planck-Institut für Radioastronomie, Bonn, 53121, Germany; 31 School of Physics & Astronomy, Cardiff University, Cardiff, Wales, CF24 3AA, UK; 32 Division of Geological and Planetary Sciences, California Institute of Technology, Pasadena, California, 91125, USA; 33 Rosseland Centre for Solar Physics, University of Oslo, Oslo, 0315, Norway

**Keywords:** galaxy clusters, intracluster medium, intergalactic medium, galaxy halos, cosmic background radiation, submillimeter facility

## Abstract

An omnipresent feature of the multi-phase “cosmic web” — the large-scale filamentary backbone of the Universe — is that warm/hot (≳ 10 ^5^ K) ionized gas pervades it. This gas constitutes a relevant contribution to the overall universal matter budget across multiple scales, from the several tens of Mpc-scale intergalactic filaments, to the Mpc intracluster medium (ICM), all the way down to the circumgalactic medium (CGM) surrounding individual galaxies, on scales from ∼ 1 kpc up to their respective virial radii (∼ 100 kpc). The study of the hot baryonic component of cosmic matter density represents a powerful means for constraining the intertwined evolution of galactic populations and large-scale cosmological structures, for tracing the matter assembly in the Universe and its thermal history. To this end, the Sunyaev-Zeldovich (SZ) effect provides the ideal observational tool for measurements out to the beginnings of structure formation. The SZ effect is caused by the scattering of the photons from the cosmic microwave background off the hot electrons embedded within cosmic structures, and provides a redshift-independent perspective on the thermal and kinematic properties of the warm/hot gas. Still, current and next-generation (sub)millimeter facilities have been providing only a partial view of the SZ Universe due to any combination of: limited angular resolution, spectral coverage, field of view, spatial dynamic range, sensitivity, or all of the above. In this paper, we motivate the development of a wide-field, broad-band, multi-chroic continuum instrument for the Atacama Large Aperture Submillimeter Telescope (AtLAST) by identifying the scientific drivers that will deepen our understanding of the complex thermal evolution of cosmic structures. On a technical side, this will necessarily require efficient multi-wavelength mapping of the SZ signal with an unprecedented spatial dynamic range (from arcsecond to degree scales) and we employ detailed theoretical forecasts to determine the key instrumental constraints for achieving our goals.

## Plain language summary

The matter content of the Universe is organized along a large-scale filamentary “cosmic web” of galaxies, gas, and an unseen “dark matter” component. Most of the ordinary matter exists as a diffuse plasma, with temperatures of 10−100 million degrees. The largest concentrations of such gas are found within gigantic galaxy clusters at the intersections of the cosmic web, weighing as much as 100 trillion Suns. As such, their masses, number and distribution across cosmic time probe the evolution and composition of the Universe itself. Also, the thermodynamics of the warm/hot gas provides an archeological record of those mechanisms that, over time, influence the large-scale structures — from the energy deposition by super-massive black holes to the collisions of massive clusters.

The best option for studying the cosmic warm/hot gas, especially at the beginnings of their formation, is provided by the so-called Sunyaev-Zeldovich (SZ) effect — a faint distortion of the Cosmic Microwave Background (CMB) observable at (sub)millimeter wavelengths. Using the SZ effect to study cosmic thermal history however requires technical advances not met by state-of-the-art (sub)millimeter telescopes. In fact, many key questions on the co-evolution of the warm/hot gas, the embedded galaxies, and the cosmic web remain unanswered.

With these motivations in mind, we discuss the development of the Atacama Large Aperture Submillimeter Telescope (AtLAST). Thanks to its unprecedented combination of a 50-meter aperture and wide 2° field of view (4× wider than the full Moon), AtLAST will map vast sky areas and detect extremely faint signals across multiple wavelengths. Overall, AtLAST will push these studies beyond the legacy of the many (sub)millimeter facilities that, during the 50 years since the theoretical foundations of the SZ effect, have pioneered the exploration of the warm/hot Universe through the SZ effect.

## 1 Introduction: clusters and the evolution of large scale structure

Clusters of galaxies, groups, and massive galaxies trace the large scale structure of the Universe, and have therefore, since their discovery, served as probes of cosmology ( Kravtsov & Borgani, 2012). For example, clusters provided the first tentative hints of dark matter ( Zwicky, 1933) as well as early evidence that we live in a universe with a low matter density Ω _*M*_ ∼ 0.2−0.3 ( Bahcall & Cen, 1992; White *et al.*, 1993). While the large catalogs compiled by cluster and large scale structure surveys have offered the testbeds of, for example, the growth of structure and cosmic shear, these tests are limited by systematics originating primarily from astrophysical effects—shocks, feedback, non-thermal pressure, and the objects’ dynamical and virialization states, to name a few — as well as from contamination due to interlopers and sources within the systems that can bias our measurements and any resulting cosmologically relevant observable (e.g., the cluster mass, a key proxy of structure evolution).

The same sources that can contaminate measurements, primarily radio-loud active galactic nuclei (AGN) and star forming galaxies, or cause departures from thermal equilibrium, such as shocks, are also the main drivers of the physical and thermodynamic evolution of the intracluster medium (ICM). The ICM, in turn, is the large scale environment within which a large fraction of galaxies reside, so the feedback and interactions between the two are important to both cosmology as well as cluster and galaxy evolution. The study of galaxy clusters is thus complex and multifaceted, but the reward to understanding the nature of clusters and large scale structure is that it allows us to test the properties of the ubiquitous dark matter, and to peer into the dark universe itself.

**Figure 1.**
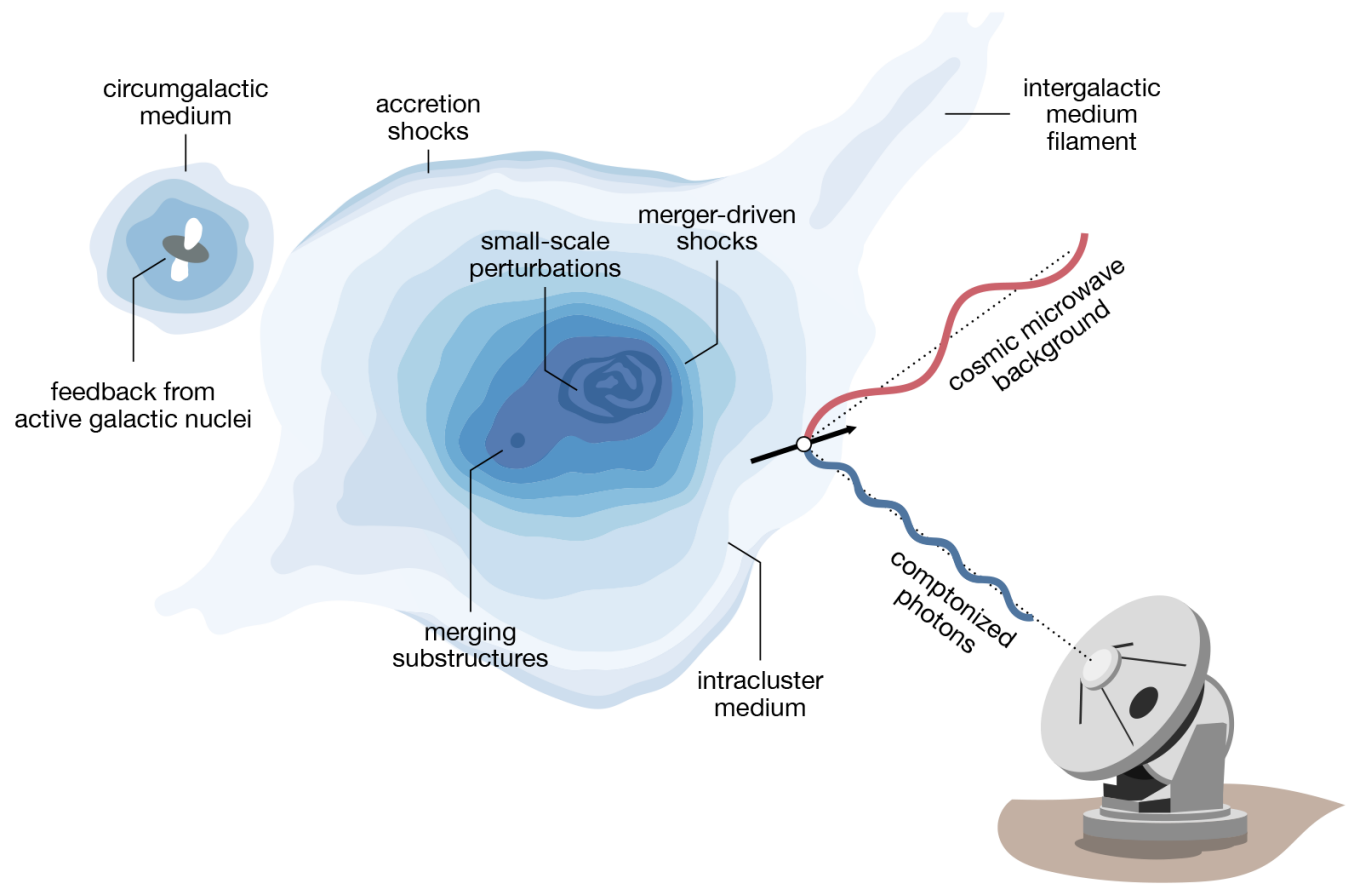
Expanded diagram highlighting some of the aspects of galaxy clusters and large-scale structures that will be studied through the Sunyaev-Zeldovich (SZ) effect using AtLAST. The SZ effect is caused by the interaction of photons from the cosmic microwave background (CMB) with reservoirs of energetic electrons within cosmic large-scale structures. Thanks to AtLAST’s unparalleled capabilities, it will be possible to fully exploit multiple aspects of the SZ effect to characterize the impact of active galactic nuclei (AGN) on the circumgalactic medium (CGM), and the multi-scale properties of the intracluster medium (ICM) of the large-scale filaments of intergalactic medium (IGM). The figure is an adaptation of the SZ schematic in Mroczkowski *et al.* (2019), which was based on that from L. van Speybroeck as adapted by J. E. Carlstrom.

Some of the dominant astrophysical processes occurring in clusters are shown in the cartoon depiction in Figure 1. Studying in greater detail the multi-scale physical processes taking place within the most massive structures in the Universe will ultimately allow us to build a more complete understanding of the thermal history of our universe, how structure grew and evolved, or fundamentally how the Universe came to be the way it is.

Here we seek to motivate deep, multi-band or multichroic high resolution and wide field observations with the Atacama Large Aperture Submillimeter Telescope ( AtLAST; Klaassen *et al.*, 2020; Mroczkowski *et al.*, 2023; Mroczkowski *et al.*, 2024; Ramasawmy *et al.*, 2022) that will address questions of cluster astrophysics as well as the contamination that could potentially plague cluster cosmology done at arcminute resolutions. At the same time, the observations discussed here are not simply to aid cosmological studies, but can probe interesting astrophysics and solve important questions about astrophysics in their own right — with the unique potential of providing a link between galaxy evolution, large-scale structure, and cosmological studies. Our primary tool here is the Sunyaev-Zeldovich effect, described below.

## 2 The multi-faceted Sunyaev-Zeldovich effect

The thermal Sunyaev-Zeldovich (SZ) effect ( Sunyaev & Zeldovich, 1970; Sunyaev & Zeldovich, 1972) — an up-scattering to higher energies of photons from the cosmic microwave background (CMB) by hot electrons — was proposed theoretically as an alternative way to probe the pressure along the line of sight (l.o.s.) of the hot gas in galaxy clusters. A schematic picture for the scattering due to the thermal SZ effect, which scales as the Compton *y* parameter ( *y* ∝ *P*_e_ *dl* for electron pressure *P*_e_ and l.o.s. *l*), is shown in Figure 1. Shortly after the theoretical foundations of the thermal SZ effect, the kinetic SZ effect (∝ *n*_e_ *v _z_dl* for electron density *n*_e_ l.o.s. peculiar velocity *v _z_*; see Sunyaev & Zeldovich, 1980) was proposed as a way to measure gas momentum with respect to the CMB, our ultimate and most universal reference frame. The following decade saw developments in the theory regarding relativistic corrections to the thermal SZ and kinetic SZ effects as well as anticipating more exotic SZ effects from non-thermal and ultrarelativistic electron populations (e.g., Enßlin & Kaiser, 2000; Itoh *et al.*, 1998; Nozawa *et al.*, 1998). Observations of the SZ effects, however, took longer to come to the fore, beginning with pioneering measurements such as Birkinshaw *et al.* (1984) and culminating more recently in several thousand measurements or detections from both low-resolution (1-10′) SZ surveys (e.g., Carlstrom *et al.*, 2011; Swetz *et al.*, 2011) and dedicated observations, often at higher (subarcminute) resolution (e.g., Adam *et al.*, 2014; Kitayama *et al.*, 2016; Mason *et al.*, 2010; Plagge *et al.*, 2013). For more comprehensive reviews of the various aspects of the SZ effect, see, e.g., Birkinshaw (1999); Carlstrom *et al.* (2002); Kitayama (2014), and Mroczkowski *et al.* (2019).

The proposed millimeter/submillimeter facility, AtLAST, presents novel, unique capabilities that will revolutionize both deep targeted observations aiming for detailed astrophysical studies, as well as wide-field surveys aiming to push SZ observations to much lower mass limits and higher redshifts. Since the epoch of reionization, the majority of baryons have been making their way up to high enough temperatures (> 10 ^5^ K) that their emission is nearly completely undetectable at optical wavelengths (visible and near-IR band), where the majority of telescopes operate. Such a hot phase is an omnipresent feature of the multi-phase cosmic web, representing a relevant contribution to the volume-filling matter budget on multiple scales — from Mpc-scale filaments of intergalactic medium, to the intracluster medium (ICM), and down to the circumgalactic medium (CGM) surrounding individual galaxies up to their virial radius (up to few 100s of kpc). Through the SZ effect, the millimeter/submillimeter wavelength regime offers a view of this important component of galaxies and their surrounding environments (clusters, groups, filaments) — components that are largely invisible to all but X-ray and SZ instruments.

## 3 Proposed science goals

**Figure 2.**
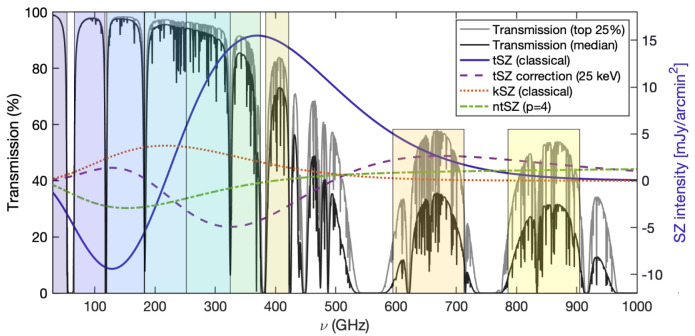
Various SZ spectra versus transmission in the top quartile (lighter gray) and median (darker gray) atmospheric transmission conditions available at the Chajnantor Plateau (≈ 5000 meters above sea level). The left y-axis corresponds to transmission, and the right y-axis is appropriate for the SZ intensity for a cluster with *y* = 10 ^−4^. The kinetic SZ values assume a line of sight velocity component *v _z_* = −1000 km/s (i.e. toward the observer, implying a net blueshift in the CMB toward the cluster) and an electron opacity *τ* = 0.01. Here we used SZpack to solve for the SZ spectral distortions ( Chluba *et al.*, 2012), and the am code for atmospheric transmission ( Paine, 2019). The optimal continuum bands of the proposed AtLAST SZ observations, reported in Table 1, are shown as background shaded regions.

Here we provide a summary of the main applications in the context of SZ studies enabled by AtLAST that will allow us to develop a more profound and complete understanding of the thermal history of the Universe, ultimately transforming our understanding of the numerous processes involved in structure formation, evolution, feedback, and the quenching of star formation in overdense environments. We refer to Lee *et al.* (2024) and van Kampen *et al.* (2024) for companion AtLAST case studies focused on emission line probes of the cold circumgalactic medium (CGM) of galaxies and on providing a comprehensive survey of high- *z* galaxies and protoclusters, respectively. Common to all the specific science cases discussed below is the need for a wide field, high angular resolution facility able to optimally probe the full SZ spectrum ( Figure 2). We refer to Section 4 for a more extended discussion of the technical requirements for the proposed science goals.

**Table 1.** Frequencies, sensitivities and beam sizes for an AtLAST type of SZ experiment. The sensitivity levels are computed assuming the standard values for weather condition (2 ^nd^ octile) and elevation ( *α* = 45 deg), but consider the broad-band re-implementation of the AtLAST sensitivity calculator. The specific frequencies of the band edges correspond to the ones that minimizes the output noise RMS level in the corresponding band per given integration time.

band —	ref. frequency [GHz]	bandwidth [GHz]	band edges [GHz]	beam [arcsec]	sensitivity [ *µ*Jy beam ^−1^ h ^1/2^]	survey noise [ *µ*K _cmb_ − arcmin h ^1/2^]
2	42.0	24	30–54	35.34	6.60	2.40
3	91.5	51	66–117	16.22	6.46	1.27
4	151.0	62	120–182	9.83	7.14	1.21
5	217.5	69	183–252	6.82	9.22	1.86
6	288.5	73	252–325	5.14	11.91	3.71
7	350.0	50	325–375	4.24	23.59	12.26
8	403.0	38	384–422	3.68	39.98	34.70
9	654.0	118	595–713	2.27	98.86	1.67 × 10 ^3^
10	845.5	119	786–905	1.76	162.51	3.70 × 10 ^4^

### 3.1 Thermodynamic properties of the ICM: radial profiles and small-scale perturbations

The morphological and thermodynamic properties of the ICM represent key records of the many physical processes shaping the evolution of galaxy clusters and groups. Non-gravitational processes — e.g., cooling, AGN feedback, different dynamical states and accretion modes ( Battaglia *et al.*, 2012; Ghirardini *et al.*, 2019) — are expected to leave their imprint on the pressure distribution of the ICM in the form of deviations from the radial models derived under universal and self-similar assumptions for structure formation (see, e.g., Arnaud *et al.*, 2010; Nagai *et al.*, 2007; Sayers *et al.*, 2023). On cluster scales, shock fronts induced by cluster mergers as well as cosmological accretion deposit their kinetic energy into the ICM, contributing to its overall thermalization ( Ha *et al.*, 2018; Markevitch & Vikhlinin, 2007). On smaller scales, turbulent motion ( Khatri & Gaspari, 2016; Romero *et al.*, 2023; Schuecker *et al.*, 2004) can induce significant non-thermal contributions to the ICM pressure support, in turn hampering the validity of the hydrostatic equilibrium assumption. We thus need robust constraints on the level of turbulence affecting the energy budget of the ICM along with an independent census of the “hydrostatic mass bias” (e.g., Biffi *et al.*, 2016) via a combination of fluctuations and resolved hydrostatic mass information. This will be crucial for inferring corrections to the hydrostatic mass due to the non-thermalized gas (see, e.g., Angelinelli *et al.*, 2020; Ettori & Eckert, 2022) and therefore strengthening the role of thermodynamic quantities for cosmological purposes ( Pratt *et al.*, 2019).

The thermal SZ effect provides a direct proxy for the (thermal) pressure due to the free electrons in the ICM and, as such, the optimal tool for gaining a direct calorimetric view of the gas thermal properties. In fact, observational models for a statistically relevant sample of clusters are currently limited to the indirect determination of resolved pressure models for clusters up to *z* ≲ 1 ( Arnaud *et al.*, 2010; McDonald *et al.*, 2014; Sayers *et al.*, 2023). Direct constraints of the properties of the ICM within protoclusters and clusters early in their formation have been obtained for only a handful of extreme systems ( *z >* 1; Andreon *et al.*, 2021; Andreon *et al.*, 2023; Brodwin *et al.*, 2016; Di Mascolo *et al.*, 2023; Gobat *et al.*, 2019; Tozzi *et al.*, 2015; van Marrewijk *et al.*, 2023) or limited samples (e.g., Ghirardini *et al.*, 2021b). Despite the significant time investment with the Atacama Large Millimeter/Submillimeter Array (ALMA; Wootten & Thompson, 2009) or the 100-meter Green Bank Telescope (GBT; White *et al.*, 2022), these observations only allow one to perform a characterization of the physical and thermodynamic state of these early systems for a few select systems. Still, these have generally required the combination with ancillary X-ray observations, due to observational limitations including poor signal to noise or the data being limited to fewer than 5 bands.

**Figure 3.**
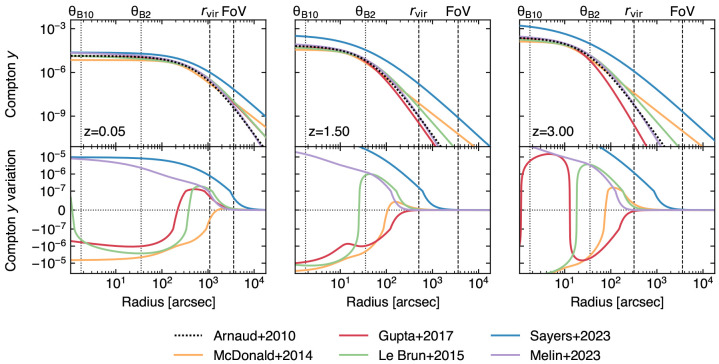
Observed Compton *y* profiles when considering different fiducial models for the ICM pressure distribution (top panels; Arnaud *et al.*, 2010; Gupta *et al.*, 2017; Le Brun *et al.*, 2015; McDonald *et al.*, 2014; Melin & Pratt, 2023; Sayers *et al.*, 2023) and respective variations (bottom panels) with respect to the universal pressure profile from Arnaud *et al.* (2010), commonly adopted as reference model for the inference of cluster masses. In this plot, we consider a cluster with fixed mass ( *M*_500_ = 10 ^14^ M _⊙_) for an arbitrary set of redshifts ( *z* = {0.05, 1.50, 3.00}). The different profiles are computed on heterogeneous samples in terms of mass and redshift ranges, and thus encode different biases associated to the intrinsic scatter of pressure profiles, deviations from self-similar evolution and hydrostatic equilibrium. Thanks to AtLAST’s sensitivity to Compton *y* levels ≲ 10 ^−7^, it will be possible to characterize such effects, while providing a model for the evolution of ICM pressure across cosmic history. As reference, we report as dashed vertical lines the virial radius of the model clusters and the instantaneous field of view expected for AtLAST (see Section 4). We further denote as dotted vertical lines the largest and smallest angular resolution *θ _i_* achievable with AtLAST, respectively obtained in the proposed Band 2 and Band 10 (see Section 4.1 and Table 1 below).

In order to gain a radially resolved view of pressure profiles and of their small-scale perturbations for a large variety of clusters (in terms of dynamical state, mass, and redshift; see Figure 3), it is key to have simultaneous access to enhanced sensitivity, high angular resolution, and wide spectral coverage across the millimeter/submillimeter spectrum. These observations are important, as from hydrodynamical simulations the pressure distribution of high- *z* galaxy clusters are predicted to diverge from the universal pressure models ( Battaglia *et al.*, 2012; Gupta *et al.*, 2017), leading to a systematic offset between the mass-to-SZ observable scaling relation for high- *z* haloes with respect to local ones ( Yu *et al.*, 2015). Constraining such deviations is crucial as they carry fundamental information on the complex interplay between all those multi-scale processes — e.g., merger and accretion events, AGN and stellar feedback, turbulent motion — at epochs ( *z >* 1) when their impact from galactic to cluster scales are expected to be the strongest. At the same time, tracing the pressure profiles out to the cluster outskirts will be key to pinpoint and characterize virial and accretion shocks ( Anbajagane *et al.*, 2022; Anbajagane *et al.*, 2024; Hurier *et al.*, 2019), whose existence is a fundamental prediction of the current paradigm of large-scale structure formation (e.g., Ryu *et al.*, 2003; Zhang *et al.*, 2021). In particular, the location and properties of their SZ features can be exploited to study the mass assembly of galaxy clusters and to place direct constraints on their mass accretion rate (a quantity otherwise difficult to infer observationally; see, e.g., Baxter *et al.*, 2021; Baxter *et al.*, 2024; Lau *et al.*, 2015; Molnar *et al.*, 2009; Towler *et al.*, 2024). And finally, pressure perturbations due to the turbulent motion within the ICM have been measured to result in fluctuations of the Compton *y* signal with fractional amplitude ≲ 10 ^−1^ compared to the underlying bulk SZ signal ( Khatri & Gaspari, 2016; Romero *et al.*, 2023). The enhanced sensitivity and calibration stability that will be achieved by AtLAST will allow it to easily probe this level of fluctuations, providing important albeit indirect information on the level of non-thermal pressure support in the ICM. More in general, it is only with the unique technical prospects offered by AtLAST that we will be able to probe to thermal SZ signal down to the levels Compton *y* ≈ 10 ^−7^ ( Section 4.2) required to probe the full extent of the ICM pressure distribution ( Figure 3), unparalleled by any of the current or forthcoming submillimeter facilities.

### 3.2 Measuring the ICM temperature via relativistic SZ effect

The classical formulation of the thermal SZ effect relies on a non-relativistic assumption for the velocity distribution of the electron populations responsible for the SZ signal. These can however manifest velocities of the order of a few tens percent of the speed of light. Accounting for any associated special-relativistic effects introduce a temperature-dependent distortion of the SZ spectral model ( Figure 2). The resulting relativistic SZ effect thus offers a valuable (yet largely unexplored) opportunity to directly measure the temperature of ICM electrons. This represents a key ingredient for enhancing our physical models of galaxy clusters and improving their utility as cosmological probes via more accurate tuning of mass calibrations and scaling relations (e.g., Lee *et al.*, 2020; Remazeilles & Chluba, 2020). At the same time, having simultaneous access to the full ICM thermodynamics (via temperature *T*_e_, as well as pressure *P*_e_ and density *n*_e_ measurements via the combination of the relativistic and purely thermal SZ effects) offers the key chance of building a temporal census of the ICM entropy distribution ( $\propto T_{\text{e}}\text{​}\;n_{\text{e}}^{-2/3},\;\text{or}\;\propto T_{\text{e}}^{5/3}P_{\text{e}}^{-2/3}$ when considering thermodynamic quantities directly probed by the SZ effect; Voit, 2005). The many processes affecting cluster evolution — e.g., AGN and stellar feedback, injection of kinetic energy due to merger activity — are observed to modify the entropy profiles throughout the cluster volumes (e.g., Ghirardini *et al.*, 2017; Pratt *et al.*, 2010; Walker *et al.*, 2012), compared to a baseline model that includes only the non-radiative sedimentation of low-entropy gas driven by gravity ( Tozzi & Norman, 2001; Voit *et al.*, 2005). As such, the spatially resolved study of the ICM entropy distribution provides a fundamental proxy of the thermal evolution of cosmic structures as well as the specific dynamical state of galaxy clusters.

Currently, estimates of the relativistic corrections to the thermal SZ effect are limited to a few pioneering studies targeting individual systems ( Hansen *et al.*, 2002; Prokhorov & Colafrancesco, 2012) or focusing on stacking analyses ( Erler *et al.*, 2018; Hurier, 2016). Still, even in the case of individual clusters with extremely rich observational spectral coverage (see, e.g., Butler *et al.*, 2022 and Zemcov *et al.*, 2012, focusing on the well-known cluster RX J1347.5-1154), SZ-based inferences of the ICM temperature have commonly resulted in constraints with limited significance. Higher angular resolutions, such as those offered by AtLAST, will be an asset for constraining SZ temperatures. First, the higher angular resolution allows spatially-distinct foregrounds such as radio sources, dusty galaxies and the Galactic dust foreground to be accurately modelled and removed. Second, the extraction of resolved pressure and temperature profiles provides the unique opportunity of performing the physical modeling of the ICM relying solely on the SZ effect. This represents a key advantage. Although electron temperatures can be measured using X-ray data, these are roughly density-square-weighted estimates (e.g., Mazzotta *et al.*, 2004) and therefore subject to biases due to clumping (e.g., Simionescu *et al.*, 2011). Further, observations can become prohibitive at large cluster radii, due to the low X-ray emissivity, and at high redshift, due to cosmological dimming. Churazov *et al.* (2015) showed that the self-similar evolution of galaxy clusters would introduce a near independence of redshift of the X-ray luminosity at fixed cluster mass — when this is defined as the mass enclosed in the radius within which the average matter density equals some fiducial cosmic overdensity value (e.g. 500 × *ρ*_crit_). Nevertheless, we note that these considerations are valid only under the assumption that the local mass-observable scaling relations are applicable at high redshift. At the same time, both the resolved SZ signal and the respective cluster-integrated flux would still be (1 + *z*) ^3/2^ larger than the X-ray emission from the same system at a given redshift *z*.

On the other hand, since the SZ effect is characterized by a surface brightness that is inherently independent of redshift, ICM temperature constraints can in principle be derived without specific limits on the distance of the target systems. And lastly, the temperatures inferred using data on the same clusters but taken using different X-ray observatories may suffer large systematic variations due to inherent calibration differences ( Migkas *et al.*, 2024; Schellenberger *et al.*, 2015). In contrast, the SZ temperature estimate is pressure-weighted and is therefore predicted to be less biased by emission while being easier to constrain at large cluster radius due to the linear (instead of squared) dependence on density. And even in the case of low-mass (i.e., low-SZ surface brightness; see also Section 3.4) clusters for which it will not be possible to extract resolved SZ-based temperature information, the availability of deep, high angular resolution SZ observations for a large sample of systems will still allow for matching resolution with X-ray observations and to extract resolved full thermodynamic properties of the ICM (see Section 3.1 and Section 5.2.4). An exploratory study of AtLAST’s expected capabilities to measure temperature via the relativistic SZ effect is presented in Section 4.3. We refer to this for more details on the impact of the specific spectral setup on the reconstruction of the relativistic SZ effect and on the technical requirements for extending such measurements over broad ranges of cluster masses and redshifts.

### 3.3 Kinematic perspective on large scale structures

The kinetic component of the SZ effect represents a valuable tool for revealing the peculiar motion of cosmic structures. Nevertheless, its properties – namely its shape, the fact that the kinetic SZ signal is generally weaker than the thermal SZ effect ( Figure 2), and that it traces the integrated line of sight momentum – make the kinetic SZ effect somewhat elusive to measure and interpret. Further, the kinetic SZ spectral distortion is consistent with a Doppler shift of the CMB photons, making it spectrally indistinguishable from small-scale primordial CMB anisotropies.

Past targetted kinetic SZ studies (e.g., Adam *et al.*, 2017; Mroczkowski *et al.*, 2012; Sayers *et al.*, 2013; Sayers *et al.*, 2019; Silich *et al.*, 2023) have already reported direct measurements of the kinetic SZ signal due to the large-scale gas flows associated with merger events. All of these works focused on individual, relatively extreme clusters (either in terms of overall mass, dynamical state, or orientation of the merger direction with respect to the line-of-sight). The broad spectral coverage and the expected sensitivity of AtLAST, in combination with its capability of probing a high dynamic range of angular scales, will instead allow for systematically including the kinetic SZ information in the reconstruction of the thermodynamic characterization of large statistical samples of galaxy clusters and groups.

Statistical measurements of the kinetic SZ effect in disturbed and merging systems represent a crucial ingredient for cosmological studies via direct measurements of the amplitude and the growth rate of cosmological density perturbations (e.g., Bhattacharya & Kosowsky, 2007; Soergel *et al.*, 2018). They can also be used to distinguish ΛCDM from alternative cosmologies with modified gravitational forces ( Bianchini & Silvestri, 2016; Kosowsky & Bhattacharya, 2009; Mueller *et al.*, 2015). Further, correlating the velocity structure with information from facilities at other wavelengths on the baryonic and dark matter content of merging systems will represent a preferential probe of the collisional nature of dark matter ( Silich *et al.*, 2023). In the case of relatively relaxed systems (i.e., with velocity fields not manifesting complex morphologies), the joint analysis of the thermal and kinematic SZ effects would naturally complement the inference of the ICM pressure and temperature distributions with information on the bulk peculiar velocity of galaxy clusters and tighter constraints on the ICM density ( Mroczkowski *et al.*, 2019).

**Figure 4.**
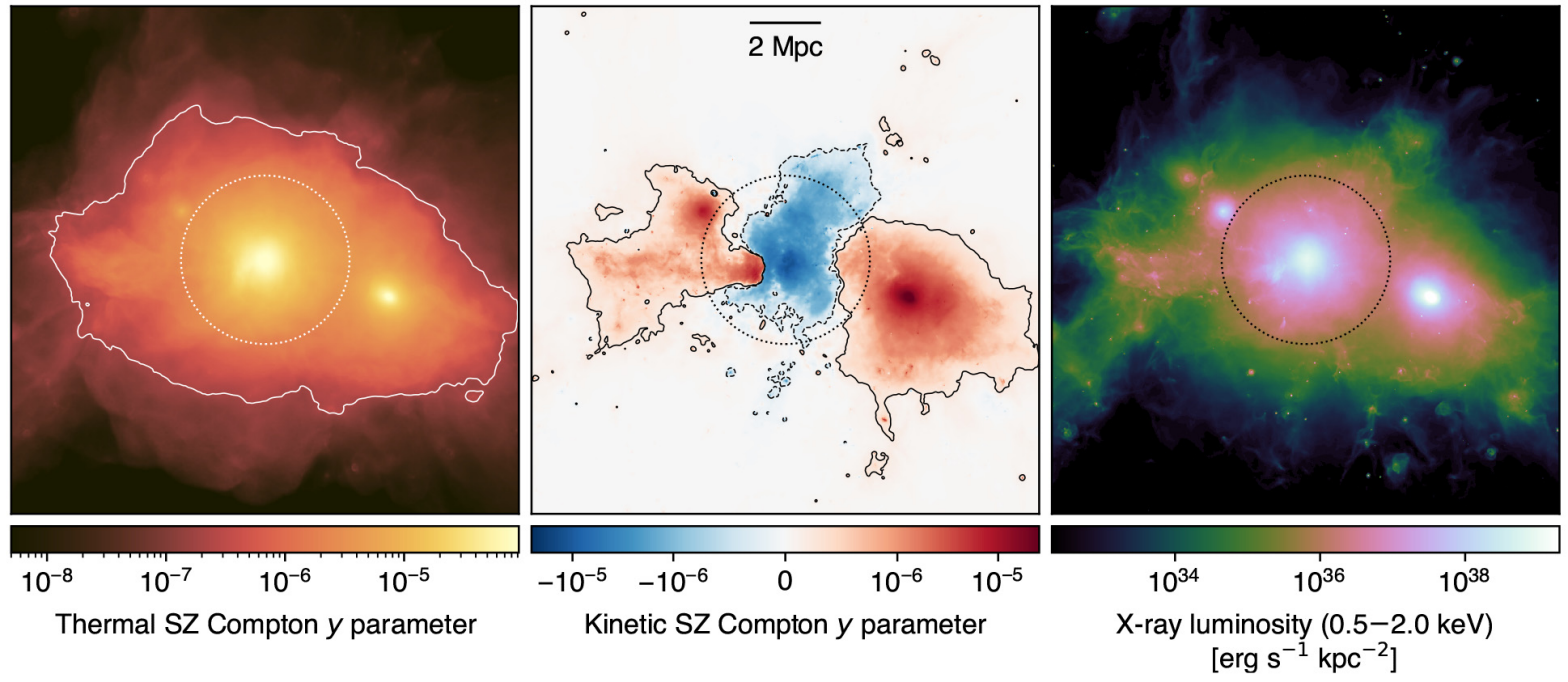
Thermal (left) and kinetic (center) SZ effects, and X-ray luminosity (right) from a simulated massive cluster undergoing a major merger ( *M*_200_ *≃* 1.5 × 10 ^15^ M _⊙_, *z* = 0) extracted from the TNG-Cluster simulation ( Nelson *et al.*, 2023). The contours in both panels trace a Compton *y* level of 2 × 10 ^−7^, roughly corresponding to the reference SZ depth for a deep AtLAST survey ( Section 4.2). As a reference, we mark with a circle the virial radius of the galaxy cluster. This implies that AtLAST will be able to efficiently trace the SZ signal out to the low-density outskirts of clusters.

The detailed spatial mapping of the kinetic SZ effect could also be used to characterize turbulent motions and to identify their driving dissipation scales which are relevant for feedback mechanisms. This can be done, in particular, by computing the velocity structure function (VSF), defined as the average absolute value of the line of sight velocity differences as a function of projected scale separation. The VSF is an effective way of characterizing turbulent motions and identifying their driving and dissipation scales (see, e.g., Ayromlou *et al.*, 2023; Ganguly *et al.*, 2023; Gatuzz *et al.*, 2023; Li *et al.*, 2020). Determining the driving scale of turbulence would constrain the relative importance of gas motions driven by AGN feedback on small scales and mergers on large scales, while the dissipation scale is sensitive to the microphysics of the ICM, such as its effective viscosity ( Zhuravleva *et al.*, 2019). In general, constraints on the small-scale properties of the velocity field associated with turbulent motion ( Nagai *et al.*, 2003; Sunyaev *et al.*, 2003), coherent rotation of gas within their host dark matter haloes ( Altamura *et al.*, 2023; Baldi *et al.*, 2018; Bartalesi *et al.*, 2024; Baxter *et al.*, 2019; Cooray & Chen, 2002), or merger-induced perturbations ( Biffi *et al.*, 2022) can complement the reconstruction of ICM thermodynamic fluctuations ( Khatri & Gaspari, 2016; Romero *et al.*, 2023) and the potential mitigation of biases due to non-thermal pressure support (e.g., Angelinelli *et al.*, 2020; Ansarifard *et al.*, 2020; Ettori & Eckert, 2022; Shi *et al.*, 2016) discussed in Section 3.1. Perturbations in the kinetic SZ distribution will result in smallscale kinetic SZ fluctuations more than an order of magnitude smaller than the corresponding thermal SZ component ( Biffi *et al.*, 2022; Mroczkowski *et al.*, 2019; Sunyaev *et al.*, 2003) even for massive systems. The clear requirement of extremely demanding observations (along with the difficulty in spectrally disentangling the kinetic SZ effect from the underlying CMB signal; Mroczkowski *et al.*, 2019) have so far limited the possibility of directly measuring any small-scale kinetic SZ feature. However, AtLAST will be able to efficiently measure percent-level deviations from the dominant thermal SZ effect (see, e.g., Section 4.3 below for a discussion in the context of relativistic SZ corrections) and to swiftly survey wide sky areas at ∼ 1.5 − 35 arcsec resolution, thus opening a novel observational window on ICM velocity substructures.

Finally, AtLAST’s simultaneous sensitivity to both small and large spatial scales facilitates studies of the distortions in the CMB across a broad range of spatial scales (300 ≲ *ℓ* ≲ 20000). Existing and forthcoming CMB experiments cannot probe beyond *ℓ* ∼ 4000, whose power spectrum is dominated by both regular CMB anisotropies and CMB lensing effects. However, at 220 GHz around *ℓ* ≈ 7000, the kinetic SZ effect becomes the dominant contributor to the angular power spectra ( Smith & Ferraro, 2017), thus enabling studies on the kinetic SZ imprint from the epoch of reionization, originating from relativistic electrons within expanding ionizing bubbles ( Ferraro & Smith, 2018) – the so-called “patchy kinetic SZ” signal.

### 3.4 Overcoming cluster selection biases

It is becoming generally appreciated that X-ray selected clusters offer a biased view of the cluster population ( Andreon *et al.*, 2017; Andreon *et al.*, 2019; Eckert *et al.*, 2011; Maughan *et al.*, 2012; Pacaud *et al.*, 2007; Planck Collaboration, 2011; Planck Collaboration 2012; Stanek *et al.*, 2006). This is because, in a given sample, bright clusters are over-represented (see, e.g., Mantz *et al.*, 2010 for discussion of Malmquist and Eddington biases), whereas those systems fainter-than-average for their mass are underrepresented, if not missing altogether. This bias is difficult to correct because the correction depends on assumptions about the unseen population ( Andreon *et al.*, 2017; Vikhlinin *et al.*, 2009). On the other hand, SZ-selected cluster samples are generally thought to offer a less biased view and indeed show a larger variety (e.g., in gas content) than X-ray selected samples (e.g., Planck Collaboration, 2011; Planck Collaboration, 2012). Comparisons of the X-ray properties of SZ-selected systems (see, e.g., CHEX-MATE Collaboration, 2021) have highlighted the fact that ICM-based selection biases can depend on the specific morphology ( Campitiello *et al.*, 2022) or the presence of a dynamically relaxed cool core (the so-called “cool-core bias”; Rossetti *et al.*, 2017).

However, the selection of clusters via their galaxies (i.e., based on the identification of cluster members) or via gravitational lensing (i.e., based on the effect of the cluster potential on the images of background sources) can provide an observational perspective that is potentially unbiased with respect to the thermodynamic state of the ICM. Although methods based on galaxies can still suffer from significant biases due to contamination and projection effects (e.g., Donahue *et al.*, 2002; Willis *et al.*, 2021), the fact that they are not dependent on the ICM-specific biases have granted the possibility of unveiling the existence of a variety of clusters at a given mass larger than X-ray or current SZ-based approaches. In particular, the low-surface brightness end of the unveiled new population of clusters is changing our view of galaxy clusters. These are found to introduce significant scatter in many ICM-based mass-observable scaling relations ( Andreon *et al.*, 2022), at very the heart of our understanding of cluster physics and broadly used in the context of cluster cosmology. Characterizing such a population of low surface brightness clusters will necessarily require a major leap in the SZ sensitivity with respect to state-of-the-art facilities.

The possibility of performing deep, high angular resolution mapping over wide sky areas offered by AtLAST will allow observers to efficiently detect those clusters that are presently underrepresented in, or entirely missing from, catalogs due to an SZ or X-ray signal inherently fainter than expected from their mass. Indeed, clusters with low X-ray surface brightness tend to have low central values of Compton *y*, of the order of few 10 ^−6^ (based on Andreon *et al.*, 2022), at the very limit of long pointed observations with current single-dish telescopes, when not beyond their effective detection capabilities. In combination with X-ray, strong and weak-lensing data, this will allow for a thorough characterization of their physical and thermodynamic state, and for discriminating between any variation in the inherent properties of the intracluster gas and observational biases induced by any astrophysical processes more or less associated with the specific evolution and physics of the target clusters — e.g., energetic AGN feedback, recent merger events, low gas fraction, enhanced clustering of millimeter-bright galaxies.

### 3.5 Identification and thermodynamic characterization of high- *z* clusters and protocluster

**Figure 5.**
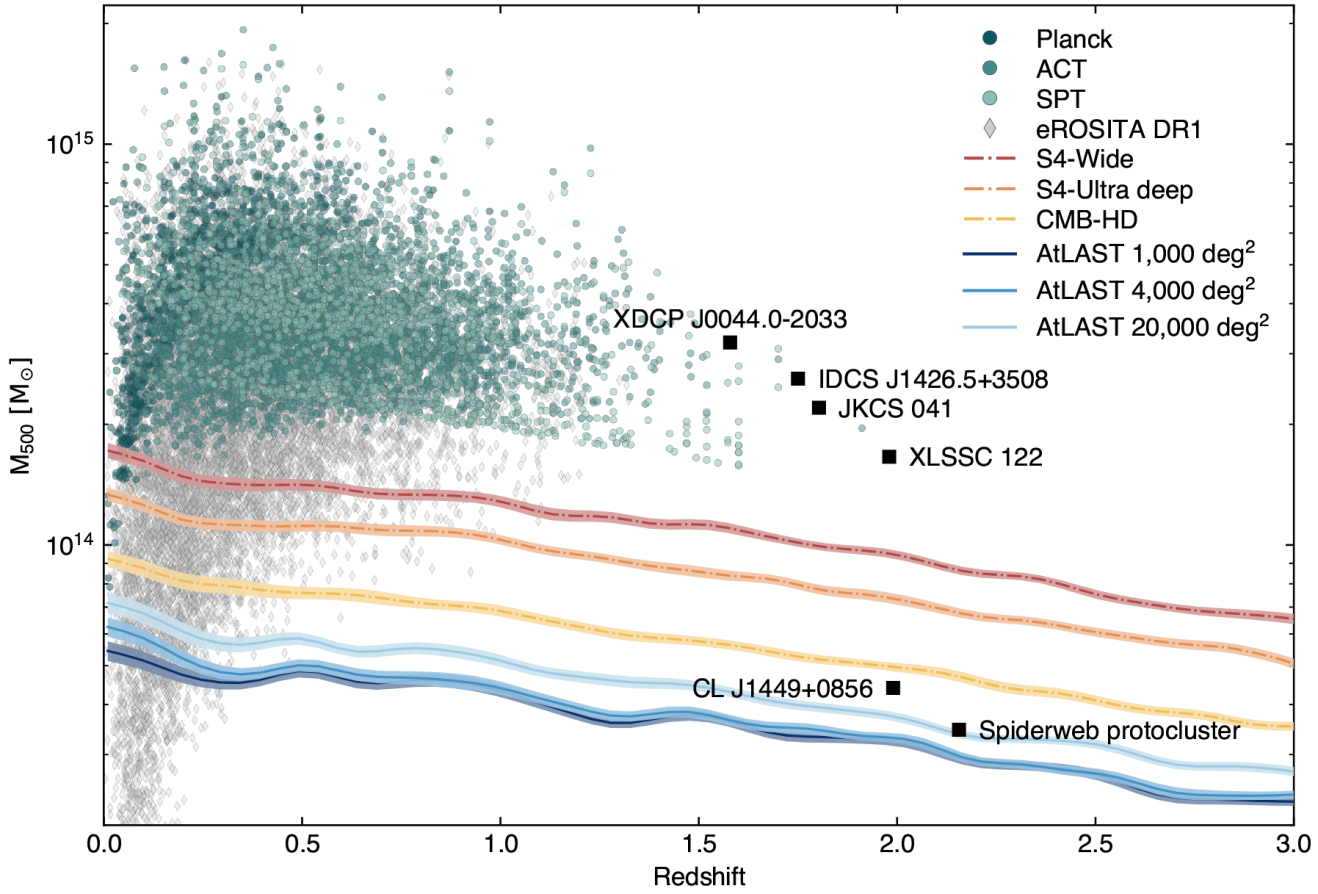
Mass vs. redshift detection forecast for AtLAST assuming different survey strategies (covering 1000 deg ^2^, 4000 deg ^2^, and 20000 deg ^2^, respectively, for a fixed survey time of 5 years) in comparison with next-generation wide-field millimeter surveys ( Abazajian *et al.*, 2016; Sehgal *et al.*, 2019) and the eROSITA all-sky (X-ray) survey ( Bulbul *et al.*, 2024). Poisson realizations of the thermal SZ confusion were simulated in the AtLAST survey case, while the other resolution surveys used Gaussian realizations appropriate in the case where the lower resolution and sensitivity limit the ability to surpass the thermal SZ confusion limit. We refer to Raghunathan (2022) for a more general discussion of the different treatments of the SZ confusion noise. For comparison, we report as green points the clusters from the available SZ survey samples ( Bleem *et al.*, 2020; Bleem *et al.*, 2024; Hilton *et al.*, 2021; Planck Collaboration, 2016), as well as relevant high- *z* clusters from the literature: XDCP J0044-2033 ( Tozzi *et al.*, 2015), IDCS J1426.5+3508 ( Brodwin *et al.*, 2016), JKCS 041 ( Andreon *et al.*, 2023), XLSSC 122 ( Mantz *et al.*, 2020; van Marrewijk *et al.*, 2023), CL J1449+0856 ( Gobat *et al.*, 2019), and the Spiderweb protocluster ( Di Mascolo *et al.*, 2023). This figure is adapted from Raghunathan *et al.* (2022).

Next generation SZ facilities like Simons Observatory (SO; Simons Observatory Collaboration, 2019) and CMB-S4 ( Abazajian *et al.*, 2016) will extend our observational window into the high- *z* and low-mass realm (see, e.g., Raghunathan *et al.*, 2022 and Figure 5) of galaxy clusters and protoclusters. Tracing the earliest phases of their evolution will be crucial for constraining the physical origin of the thermal properties of the large-scale structures observed in the nearby Universe.

Nevertheless, current forecasts estimate that next-generation wide-field surveys ( Gardner *et al.*, 2024) will detect less than 20% of the most massive (proto)clusters ( *M*_200_ ≲ 10 ^14^ M _⊙_, *z* > 2). This is mostly a consequence of the competing impact of inherently low SZ amplitudes (due to low mass, disturbed state, and severe deviations from full gas thermalization and virialization; Bennett & Sijacki, 2022; Li *et al.*, 2023; Sereno *et al.*, 2021), the low angular resolution of the facilities, and of the increasing contamination level due to, e.g., enhanced star formation and AGN activity, or possibly due to massive CGM gas and dust reservoirs at high redshift ( Lee *et al.*, 2024). And as already broadly discussed in Section 3.1, extreme limitations are also faced in the case of high angular resolution measurements. Clearly, having access to deep, high angular resolution and multi-band SZ observations will allow observers to simultaneously tackle all such issues, making AtLAST the optimal telescope that will definitively shape our perspective on high- *z* (proto)clusters.

**Figure 6.**
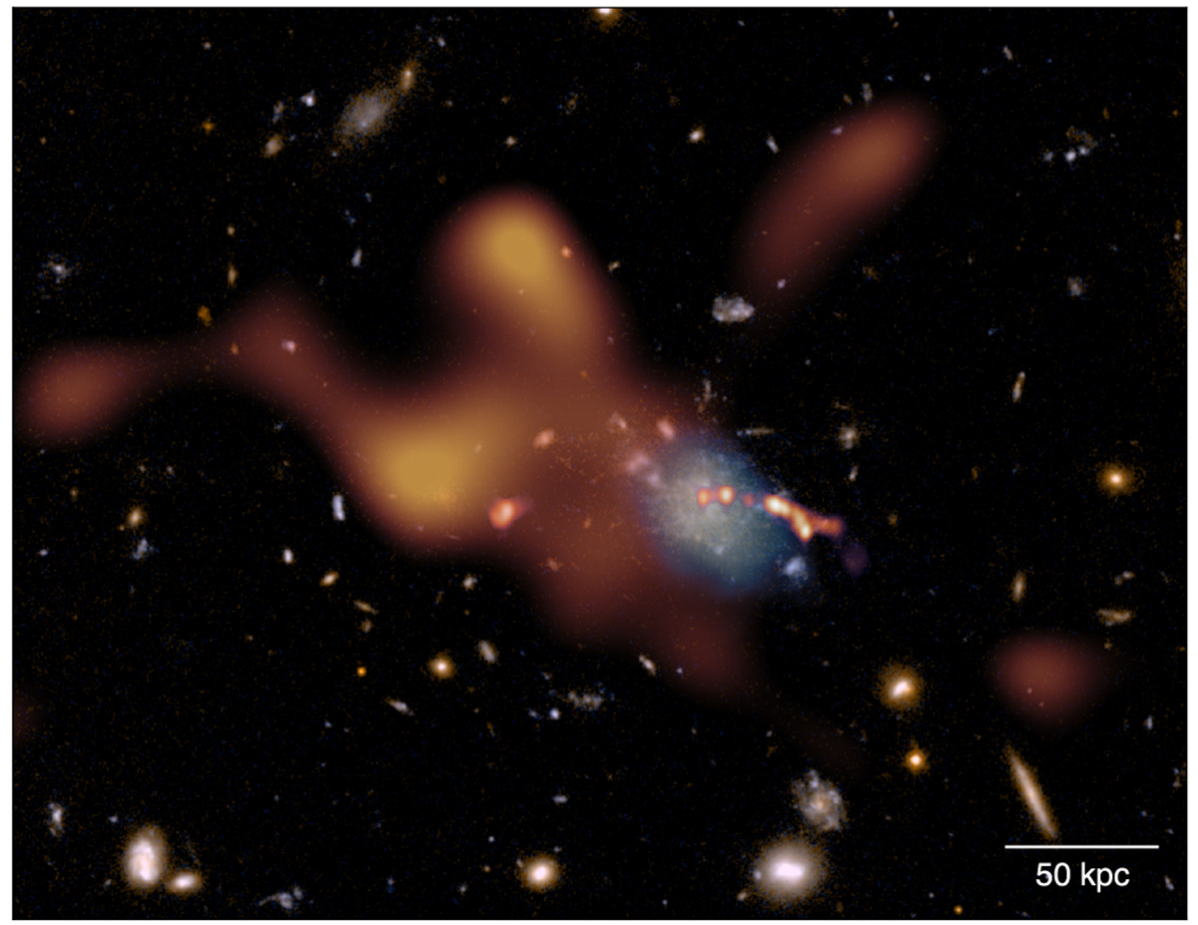
Composite Hubble Space Telescope (HST) image based on ACS/WFC F475W and F814W data of the Spiderweb protocluster field. Overlaid (orange) is the thermal SZ signal from the ICM assembling within the protocluster complex as observed by ALMA over a total of more than 12 h of on-source integration time. In a similar amount of time and with the same spectral tuning, AtLAST will achieve a depth comparable to ALMA, however providing a dramatic improvement of ∼ 10 ^3^ in field of view and, thus, in overall mapping speed. At the same time, AtLAST will provide a novel perspective on the reservoirs of cold gas (light blue overlay; Emonts *et al.*, 2016) coexisting with the warm/hot phase within protocluster cores (see the CGM science case study by Lee *et al.* (2024) for a discussion). For comparison, we also include the bright jet of radio emission output from the central galaxy as observed by VLA (the linear east-west feature, shown in red; Carilli *et al.*, 2022). The present figure is adapted from Di Mascolo *et al.* (2023) and the corresponding ESO Press Release eso2304.

The correlation of such constraints with the properties of the galactic populations observed within the (proto)cluster complexes will further allow for directly linking the evolution of the forming intracluster gas to the multi-phase protocluster environment and its only partially understood impact on galaxy formation and evolution ( Figure 6). Current multi-wavelength observations have highlighted that the environmental effects might act on Mpc scales and well beyond the more or less virialized regions within these protocluster galaxy overdensities ( Alberts & Noble, 2022). These studies however rely on the characterization of environmental processing solely from the perspective of protocluster galaxies ( Overzier, 2016). On the other hand, the wide field, the extreme sensitivity and the capability of AtLAST to trace low density regions thanks to the SZ effect will allow for an efficient imaging of the complex galaxy-environment puzzle with a comprehensive glance of the multi-scale and multi-phase nature of high- *z* (proto)cluster systems.

### 3.6 Impact of AGN feedback and halo heating

Acting in practice as a calorimeter of astrophysical electron populations, the thermal SZ effect can shed light on the interplay of feedback processes and heating of large-scale halos from galactic to cluster scales. This is particularly relevant in the context of AGN studies, in relation to the specific impact of feedback and AGN-driven outflows in contributing to the heating of cosmic haloes ( Fabian, 2012). In fact, despite the importance of supermassive black holes (SMBH) in driving the evolution of cosmic structures, we still have a limited understanding of the complex connection between multi-scale physical properties of SMBH and their host galaxies ( Gaspari *et al.*, 2020). Current multi-wavelength observations support a rough duality in the feedback framework ( Padovani *et al.*, 2017), with the level of radiative efficiency depending on the specific scenario regulating SMBH accretion ( Hlavacek-Larrondo *et al.*, 2022; Husemann & Harrison, 2018). From the perspective of the observational properties of the hot ICM/CGM phase, different feedback models would naturally result in different levels of energy injection and, thus, in deviations from the halo thermal budget expected from virial considerations. At the same time, the strong interaction of winds and jets with the surrounding medium introduces a significant amount of non-thermal support to the overall pressure content — in the form, e.g., of turbulent motion, buoyantly rising bubbles of extremely hot plasma (≳ 100 keV) and associated shock-heated gas cocoons ( Abdulla *et al.*, 2019; Ehlert *et al.*, 2019; Marchegiani, 2022; Orlowski-Scherer *et al.*, 2022; Pfrommer *et al.*, 2005). All this implies, however, that gaining a detailed view of the thermodynamic properties of the circumgalactic haloes would allow us to obtain better insights into the AGN energetics and improve our feedback models.

Measurements of the integrated thermal SZ signal have already been broadly demonstrated to provide an efficient means for probing the evolution of the imprint of feedback on the thermal energy of cosmic structures ( Crichton *et al.*, 2016; Hall *et al.*, 2019; Yang *et al.*, 2022). These are however limited mostly to stacking measurements of arcminute-resolution SZ data, and are thus hampered by the low angular resolution of the wide-field survey data employed. On the other hand, targeted observations at higher angular resolution currently comprise an extremely small set of high- *z* quasars ( Brownson *et al.*, 2019; Jones *et al.*, 2023; Lacy *et al.*, 2019). The overall limited sensitivity as well as interferometric effects such as poor *uv*-coverage and the filtering of large scales, however, resulted only in what appear to be low significance detections of the SZ signal in the direction of these systems. While these works have been pioneering for high resolution studies, they so far provide little constraining power on the AGN energetics and feedback scenarios.

On the other hand, the SZ signal from AGN-inflated bubbles has been robustly detected in one, extreme case (MS 0735.6+7421; Abdulla *et al.*, 2019; Orlowski-Scherer *et al.*, 2022). Still, the observations required 10s hours with the current-generation MUSTANG-2 instrument ( Dicker *et al.*, 2014), and 100s of hours with the previous-generation CARMA interferometer ( Woody *et al.*, 2004), and were limited to single frequency observations. Since the SZ signal scales as the amount of energy displaced, future observations with current instruments to observe additional, less energetic AGN outbursts could require much more time on the source. As such, this singular example serves largely as a proof-of-principle for further, future resolved studies. We note that some progress will be made in this decade with, e.g., TolTEC ( Bryan *et al.*, 2018), though the Large Millimeter Telescope Alfonso Serran (LMT; Hughes *et al.*, 2010) was designed to achieve a surface accuracy of ∼ 50 *µ*m (2.5× worse than AtLAST), and regardless will be limited by the atmospheric transmission to *ν* ≲ 350 GHz in all but the most exceptional weather (see, e.g., the site comparison in Klaassen *et al.*, 2020). Other single dish facilities delivering similarly high resolution will be limited to even lower frequencies (e.g. Nobeyama, Green Bank Telescope, Sardinia Radio Telescope), while ALMA has difficulty recovering scales larger than 1′ in all but its lowest bands (see Section 4).

Recently, multiple studies (e.g., Chakraborty *et al.*, 2023; Grayson *et al.*, 2023; Moser *et al.*, 2022) showed that obtaining high angular resolution observations of the thermal SZ effect (in combination with X-ray observations) would allow for constraining the distinct contribution from different feedback models. First observational studies based on the cross-correlation of the thermal and kinetic SZ signals (e.g., Amodeo *et al.*, 2021; Das *et al.*, 2023; Schaan *et al.*, 2021; Vavagiakis *et al.*, 2021) already showed independent and competitive constraints. Recently, Coulton *et al.* (2024) demonstrated that the socalled “patchy screening” can provide an alternative and highly complementary perspective on feedback mechanisms. Still, the low angular resolution of such measurements is not sufficient to spatially separate first and higher-order halo terms, and are thus hampered by respective systematics. On the other hand, based on numerical predictions for different feedback models ( Yang *et al.*, 2022), extending our observational constraints to include a broad range of masses and redshift and distinguishing between different feedback models will be highly impractical with current high angular resolution facilities. Further, it is worth noting that strongly asymmetric outflows from quasars, as well as gas inflows, would result in small-scale distortions of the overall SZ signal due to the localized thermal, kinetic and relativistic SZ contributions (see, e.g., Bennett *et al.*, 2024). Similarly, the inflation of cavities by large-scale jets and the consequent generation of shock fronts and turbulent motion would imprint observable deviations in the global SZ signal in the direction of AGN hosts ( Ehlert *et al.*, 2019). Having access to sensitive, multi-frequency observations as provided by AtLAST would thus be crucial, on the one hand, for reducing any biases associated with the missing decomposition of the different SZ components to the measured signal as well as any contamination (due to, e.g., millimeter/submillimeter bright emission from the AGN within the studied haloes). On the other hand, it will allow for cleanly dissecting the spectral and morphological features characteristic of the different feedback scenarios. Concerning the reconstruction of the thermal properties of the CGM, this will have an impact even beyond the context of the evolution of the physical processes driving the heating of cosmic haloes. In fact, it will be possible to swiftly build a multi-phase picture of the CGM by concurrently tracing its cold phase along with direct constraints on the otherwise elusive warm/hot constituent — comprising ≈ 80% of the total baryonic material in the CGM overall (e.g., Schimek *et al.*, 2024). This is an unparalleled feature of (sub)millimeter measurements, that necessarily require a combination of high spectral and angular resolution, along with the capability of mapping large-scale diffuse signals. Clearly, AtLAST will be the optimal facility for such a task. For a broader discussion of the importance of multi-phase CGM studies in the context of galaxy formation and evolution, we refer to the companion AtLAST CGM science case study by Lee *et al.* (2024).

### 3.7 Galaxy cluster outskirts and intercluster structures

A significant portion of the baryonic content of the Universe at *z* ≲ 3 is expected to lie well beyond the virial boundaries of cosmic structures ( Cen & Ostriker, 1999). This diffuse “warm-hot intergalactic medium” (WHIM) is expected to have temperatures *T*_e_ ≈ 10 ^5^ − 10 ^7^ K, largely invisible at optical wavelengths and generally too low in temperature for all but the deepest X-ray observations, often being limited to line of sight absorption studies ( Nicastro *et al.*, 2018). Obtaining a detailed view of the large-scale WHIM is however crucial. Accurately constraining the actual amount of matter constituting the WHIM will provide fundamental information on the “missing baryons” budget associated to this specific phase of the filamentary intergalactic medium (e.g., Shull *et al.*, 2012). This will be connected to the specific mechanisms driving the heating of large-scale structure on cosmological scales: on the one hand, matter inflows and mergers along large-scale filaments driving strong accretion and virialization shocks ( Anbajagane *et al.*, 2022; Anbajagane *et al.*, 2024; Baxter *et al.*, 2021 see also Section 3.1); on the other hand, the impact of feedback processes and of the environmental preprocessing of galaxies (e.g., Alberts & Noble, 2022; Fujita, 2004).

**Figure 7.**
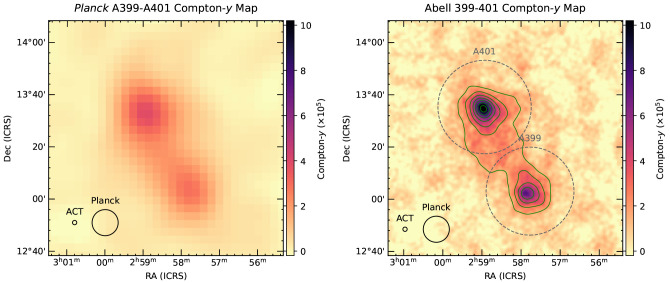
Comparison of the Compton- *y* maps produced with *Planck* alone (left, 10′ resolution) and ACT (right, 1.7′ resolution). AtLAST will deliver an 8.3× improvement in resolution, and 69.4× the instantaneous sensitivity per beam, with respect to that of ACT (and other 6-m CMB experiments) for the same observing frequencies, allowing one to image substructures in intercluster bridges and directly identify and remove source contamination. The figure has been reproduced and adapted with permission from Hincks *et al.* (2022).

To date, the identification and characterization of the physical properties of the filamentary WHIM has been performed mostly through stacked SZ and/or X-ray measurements (e.g., de Graaff *et al.*, 2019; Singari *et al.*, 2020; Tanimura *et al.*, 2019; Tanimura *et al.*, 2020; Tanimura *et al.*, 2022), and is often dominated by the hottest extremes of the range of temperatures expected for the WHIM (see Lokken *et al.*, 2023 for discussion). Recently, direct SZ imaging of a nearby intercluster bridge was presented in Hincks *et al.* (2022), which used the combination of ACT+ *Planck* data to reveal details at a much higher spatial dynamic range than the previous results using *Planck* alone. The results are shown in Figure 7. This work, while serving as a valuable pathfinder, highlighting the SZ substructures possible to image at even modestly higher (∼ 6×) resolution, was still limited to nearby ( *z* ≈ 0.05) massive clusters. Deep maps with AtLAST will allow improved spatial dynamic range and higher fidelity, enabling such studies for many more clusters going to both higher redshifts and lower mass regimes.

Thanks to its sensitivity and to the possibility of recovering large scales over extremely wide fields, AtLAST will provide the ideal tool for searching for the presence of the SZ effect in accreting and unbound intergalactic gas surrounding the virialized volume of clusters and groups. In particular, this will make it possible to routinely explore intercluster structures in a large number of cluster pairs without the need for time demanding observations. For instance, it will be possible to achieve the same Compton *y* (or surface brightness) sensitivity as in the observation of the A399-A401 observations by Hincks *et al.* ( 2022, see also Figure 7) in less than ∼ 10 h of integration, but with better spectral coverage and an order of magnitude improvement in the angular resolution. On the other hand, we can consider as a rough lower limit of the expected amplitude for large-scale filaments the results from previous stacking experiments on intergalactic gas. For instance, de Graaff *et al.* (2019) provide estimates of the average SZ signal to have amplitudes in Compton *y* unit of ≲ 10 ^−8^, corresponding to a maximum amplitude of the thermal SZ signal of ≳ −64 nJy beam ^−1^ for the decrement, and ≲ 9.5 nJy beam ^−1^ for the increment. Although impractical for performing any direct imaging of WHIM between and around individual galaxies, the extreme observing speed of AtLAST will allow for extending the stacking constraints to higher redshift and resolutions, providing a resolved evolutionary perspective on the hot phase of the cosmic web and the processes driving their thermal properties. Similarly, the broad spectral coverage will allow for reducing contamination from inter-filamentary structures, while providing the means for directly inferring robust temperature constraints (currently representing the main limitation for using the SZ effect for determining the overall contribution of WHIM to the missing baryon budget).

## 4 Technical justification

The field of view of a (sub)millimeter telescope represents a key parameter in the context of SZ science. Current high resolution instruments on large single dish telescopes — e.g., MUSTANG-2 ( Dicker *et al.*, 2014), NIKA2 ( Adam *et al.*, 2018), TolTEC ( Bryan *et al.*, 2018) — lose signals on scales larger than their instantaneous fields of view (≈ 4 − 6′; Romero *et al.*, 2020), where much of the most interesting, faint target SZ signals exist. We note that continuum observations using the 12-meter antennas in the ALMA Total Power Array (TPA; Iguchi *et al.*, 2009) suffer even more egregiously from being unable to remove atmospheric contamination due to their limited fields of view. They also suffer from the poor mapping speeds associated with single beam observations, and from relatively small collecting areas. The issue associated with large-scale filtering is arguably more restrictive in the case of interferometric observations, which generally feature maximum recoverable scales that fall within the sub-arcminute regime (e.g., ALMA Bands 4–10; we refer to the ALMA Technical Handbook for further details). So far, instruments with much larger instantaneous fields of view, which are therefore better able to recover larger scales, have been employed in the context of CMB/SZ survey experiments like ACT ( Swetz *et al.*, 2011), SPT ( Carlstrom *et al.*, 2011), SO ( Simons Observatory Collaboration, 2019), CMB-S4 ( Abazajian *et al.*, 2016), CCAT-prime (Fred Young Submillimeter Telescope or FYST; CCAT-Prime Collaboration, 2023). Still, these feature small apertures (≤ 15-m). This results in poor source sensitivity due to their limited collecting areas and in arcminute-level angular resolution, making these telescopes not suitable for imaging the small-scale morphologies of galaxy clusters and protoclusters (except for a few systems in the nearby Universe). In general, larger scales are difficult to recover due to the large, and largely common mode, atmospheric signal which dominates. A field of view of reduced size requires a tailored observational strategy and data reduction pipeline to mitigate signal loss at large scales. Nevertheless, even in such a case, the recovery of astrophysical information beyond the maximum recoverable scales of such facilities would still be severely hampered. This would critically affect many of the proposed science goals, particularly those requiring both wide field of views and extended recoverable scales. Intergalactic filaments are in fact expected to extend over tens of Mpc (e.g., Galárraga-Espinosa *et al.*, 2020) and, thus, extending over degreescales in the case of nearby superclusters ( Ghirardini *et al.*, 2021a). Similar physical extents are observed also in the case of high- *z* protocluster complexes (≲ 20 arcmin; see, e.g., Cantalupo *et al.*, 2019; Hill *et al.*, 2020; Jin *et al.*, 2021; Matsuda *et al.*, 2005). And as shown in Figure 3 and Figure 4, effectively probing the distribution of the ICM thermodynamic properties out to the cluster outskirts requires mapping the SZ signal beyond ∼ 1 deg in clustercentric distance. Therefore, the capability of gaining instantaneous observations of structures extending from few arcminutes up to degree scales will represent a crucial benefit of AtLAST compared to state-of-the-art and future telescopes covering the same observational windows.

Multi-band observations are also critical to suppress/mitigate non-SZ signals below the detection threshold, making wide spectral coverage essential for many of the science goals detailed above. Current high resolution facilities on large telescopes have at most three bands, and are limited to relatively low-frequency observations — e.g. ≤ 350 GHz for the LMT ( Hughes *et al.*, 2010), ≤ 270 GHz for the 30-meter Institute for Millimetric Radio Astronomy (IRAM), and ≤ 115 GHz for the 100-meter GBT ( White *et al.*, 2022), the 64-meter Sardinia Radio Telescope (SRT; Prandoni *et al.*, 2017), or any potential single dish component of the ngVLA ( Selina *et al.*, 2018). This implies that any current or next-generation facilities will provide limited constraining power for the relativistic and kinetic SZ, as well as contamination from the cosmic infrared background or diffuse dust contamination.

Most foregrounds should be spatially distinguishable from the SZ signal. However there may be a spatially coincident large-scale dust component originating from within clusters themselves (e.g., Erler *et al.*, 2018) which makes at least two bands in the range 400−900 GHz indispensable to trace the Rayleigh-Jeans tail of the dust spectral energy distribution and to mitigate biases in the SZ spectral modeling. An additional band at ≈ 1200 GHz would be even more helpful to resolve degeneracies between dust and SZ signals, but this is precluded by the severe reduction in atmospheric transmission.

To meet the observational requirements for pursuing the proposed scientific goals ( Section 2), we perceive the most salient instrumentation requirements to be the ability to achieve high continuum mapping speeds over large areas and in multiple bands. This would convert into the key demand of densely filling the telescope focal plane with a large count of multi-frequency detectors. In this regard, current state-of-the-art continuum cameras (e.g., transition edge sensor bolometers or kinetic inductance detectors) have already demonstrated a high technical readiness level, providing background-limited performance in the (sub)submillimeter the possibility of being read out in large numbers (tens-to-hundreds of thousands, as noted in Klaassen *et al.*, 2020) through frequency multiplexing, allowing the construction of large imaging arrays. We further refer to the AtLAST Memo 4 for details on the expected instrumental specifications for AtLAST.

**Figure 8.**
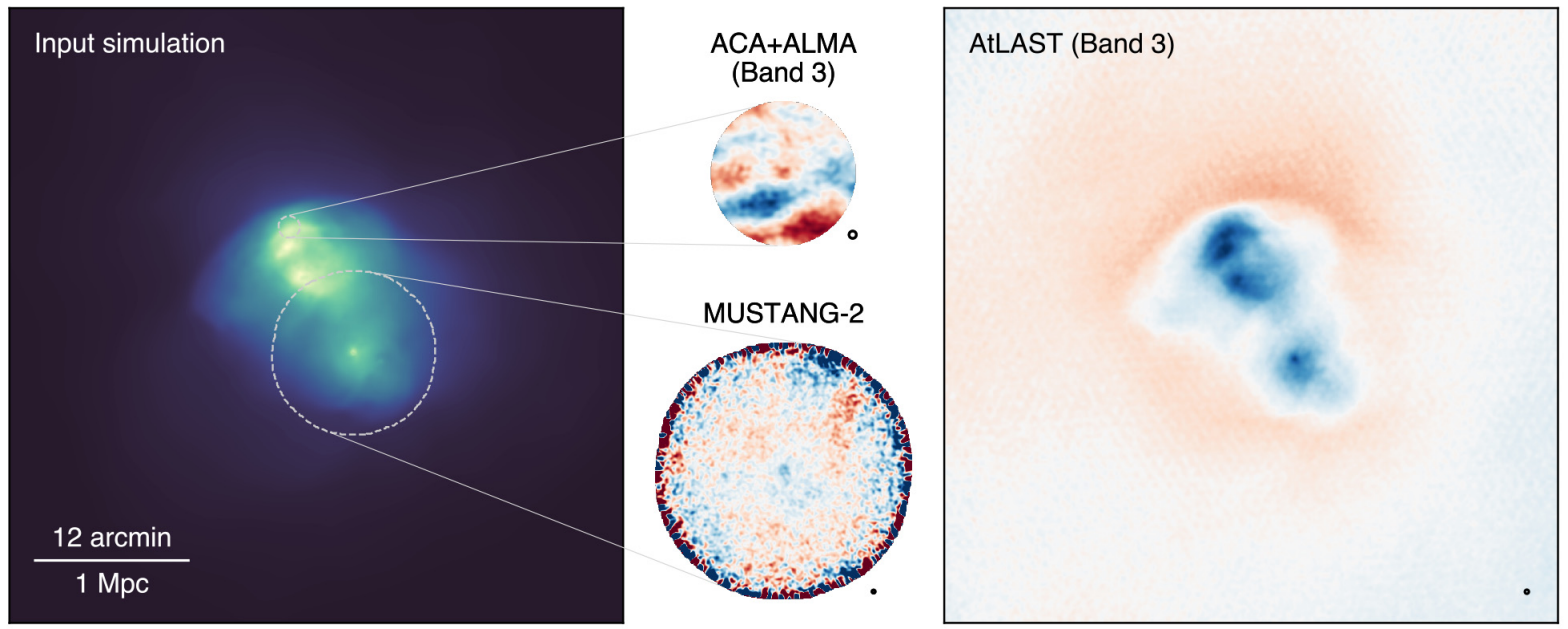
A simulated nearby galaxy clusters ( *M*_500_ = 1.28 × 10 ^15^ M _⊙_, *z* = 0.0688; left) as observed by ALMA+ACA in Band 3 (top center), MUSTANG-2 (bottom center), and by AtLAST in Band 3 (right). The respective beams are shown in the bottom right corner of each panel. The input simulation is extracted from the Dianoga cosmological simulation suite ( Bassini *et al.*, 2020; Rasia *et al.*, 2015). Overlaid as dashed white circles are the ACA+ALMA and MUSTANG-2 footprints. We note that the respective panels on the central column are scaled up arbitrarily with the goal of highlight any observed features, and do not reflect the relative angular sizes of the fields. For all cases, we consider an on-source time of 8 hours. The mock AtLAST and MUSTANG-2 observations are generated using the maria simulation tool (see van Marrewijk *et al.*, 2024 for details), assuming an AtLAST setup with the minimal detector counts of 50,000 ( Section 4.2). For ACA+ALMA, we employ the simobserve task part of the Common Astronomy Software Applications (CASA; CASA Team, 2022).

To illustrate the observational capabilities of the proposed AtLAST continuum setup, we generate mock observations using the maria simulation library ( van Marrewijk *et al.*, 2024) and consider a simulated galaxy cluster extracted from the Dianoga hydrodynamical cosmological simulations ( Bassini *et al.*, 2020; Rasia *et al.*, 2015) as input. The results are shown in Figure 8. For comparison, we include simulated observations performed with MUSTANG-2 and jointly with ALMA and the 7-m Atacama Compact Array (ACA; Iguchi *et al.*, 2009). The clear result is the superior capability of AtLAST in recovering spatial features over a broad range of scales at high significance, while MUSTANG-2 and ACA+ALMA suffer from limited sensitivity and significant large-scale filtering, respectively. We note that, in this test, we are considering only single-band observations at the same frequency to facilitate the comparison. Although ALMA Band 1 offers an improved sensitivity, spatial dynamic range, and field of view compared to Band 3, it still provides a limited sampling of largescale SZ structures (with a maximum recoverable scale MRS ≲ 1.20′ when ALMA is in its most compact configuration).

### 4.1 Optimizing the spectral setup

As mentioned broadly in Section 2 and discussed in the introduction to this section, among the critical aspects for performing a robust reconstruction of the SZ effect is the requirement of cleanly separating the multiple spectral components of the SZ signal from contaminating sources. From a technical point of view, this converts to maximizing the spectral coverage while requesting maximum sensitivity (i.e., lowest noise root-mean-square) for each of the bands. Given the deteriorating atmospheric transmission when moving to higher frequencies, this is not obtained by trivially expanding the effective bandwidth arbitrarily. At the same time, we would like to consider a minimum setup in order not to result in an over-sampling of the target spectral range.

**Figure 9.**
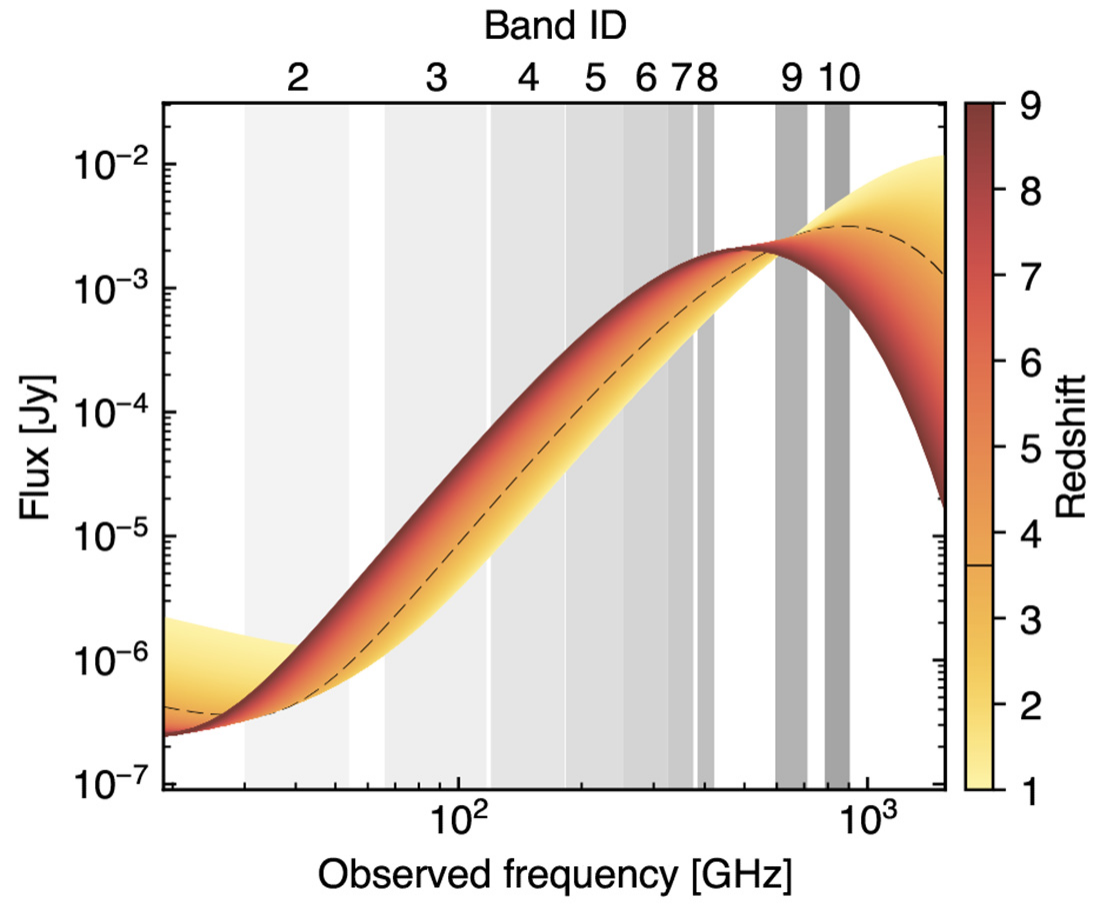
The high-frequency bands (Band 8–10) will be crucial for optimally sampling the peak of the dust continuum emission from individual high- *z* background galaxies. Along with inferring the physical properties of their dust content, this will be crucial for minimizing the contamination of the SZ signal due to cospatial dusty components (see also Section 4.3). As a reference, we show here model emission for star-forming galaxies at varying redshift. Here, the dust contribution is based on the *z*-dependent dust temperature model from Sommovigo *et al.* (2022) for a dust mass *M*_d_ = 10 ^8^ M _⊙_ (consistent with the galaxy REBELS sample; Bouwens *et al.*, 2022). The low-frequency radio component reproduces the radio model from Delvecchio *et al.* (2021), assuming an infrared-to-radio luminosity ratio of *q _IR_* = 2.646. The dashed line denotes the lowest redshift at which the dust emission peak falls within the AtLAST spectral range ( *z ≃* 3.60).

A summary of the selected bands, specifically optimized to minimize the output noise root-mean-square level per given integration time, is provided in Table 1. Our low-frequency set (≲ 300 *GHz*) extend upon the multi-band set-up proposed for CMB-S4 ( Abazajian *et al.*, 2016), shown in forecasts to provide an optimal suppression of the contribution from astrophysical foregrounds and backgrounds ( Abazajian *et al.*, 2019). Nevertheless, motivated by the expected coverage of the ≲ 30 GHz range by future radio facilities (e.g. SKA, ngVLA), we decide do not include the synchrotron-specific 20 GHz band. On the other hand, given the centrality of the high-frequency (≳ 500 GHz) for maximizing AtLAST’s capability of separating different SZ components and the signal from contaminating sources (see Section 4.3), we extend the overall spectral coverage beyond 300 GHz to include four additional bands up to 900 GHz. Compared to FYST’s choice of survey bands ( CCAT-Prime Collaboration, 2023), our choice will allow one to better sample the high-frequency end of AtLAST’s spectral range and, in turn, to gain a better handle on the relativistic SZ effect and on the dust contamination ( Figure 9; we further refer to van Kampen *et al.*, 2024 for a direct comparison of the large-scale distribution of submillimeter bright sources observed by the arcminute-resolution ACT and AtLAST). Currently, we are investigating the possibility of integrating an additional band covering the ∼ 500 GHz atmospheric window, but the low transmission and limited fractional bandwidth are expected to limit the effectiveness of such an addition in terms of an increase of the overall SZ sensitivity. However, we emphasize that AtLAST coverage of the ALMA Band 8 frequencies up to *ν* = 492 GHz would be fundamental for other application in the context of AtLAST science. We refer the interested readers to the companion AtLAST case studies by Lee *et al.* (2024) and Liu *et al.* (2024).

### 4.2 Survey strategy and detector requirements

To obtain a straightforward estimate of the instrumental performance expected when adopting the proposed spectral setup, we extend the analysis performed by Raghunathan (2022) to simulate an AtLAST-like facility (we refer to the aforementioned paper for technical details). As broadly discussed in the previous sections, performing a clean and robust separation of the multiple spectral components determining the millimeter/submillimeter sky will represent the major observational challenge to the achievement of the proposed SZ science goals. This will inherently result in more or less severe residual noise, as a combination of any contributions from instrumental noise, galactic foregrounds and extragalactic backgrounds are not properly accounted for the separation. As such, it represents a limiting factor in the detectability of any SZ signal and could be interpreted as the final SZ depth of the proposed observations.

**Figure 10.**
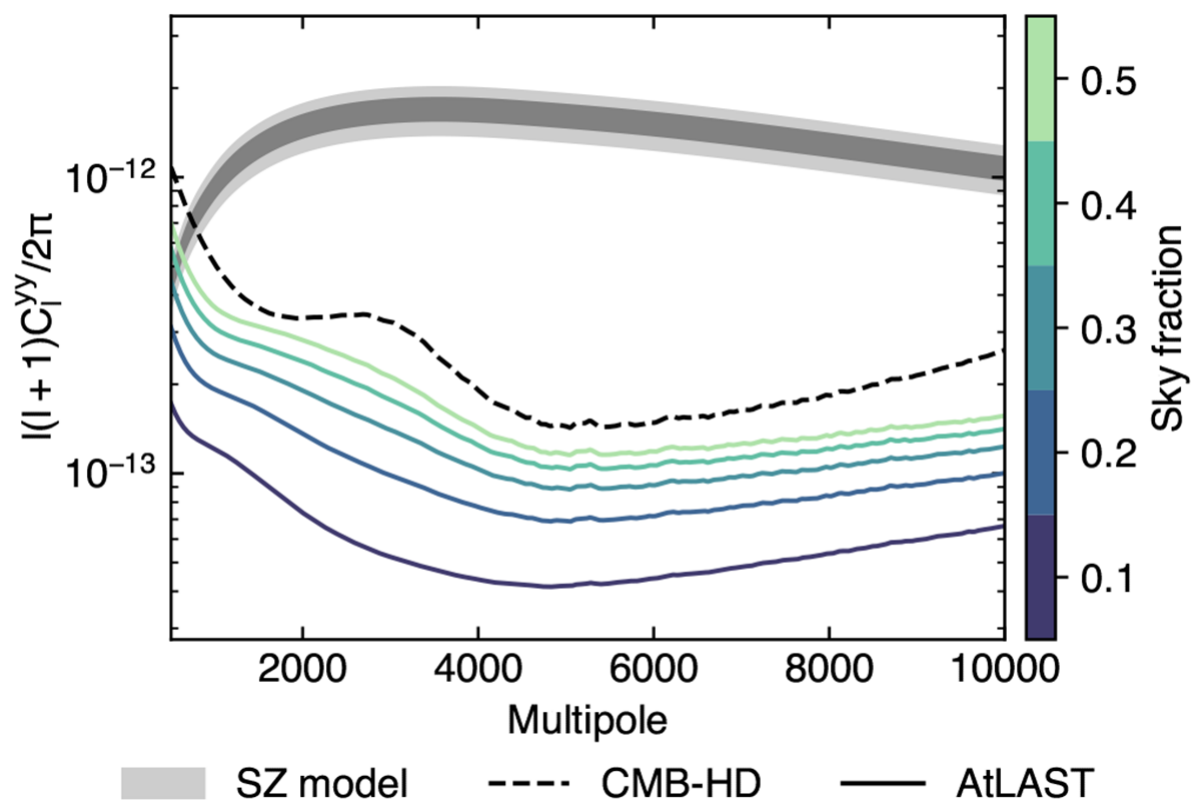
Residual Compton- *y* noise power spectra as a function of the sky coverage ( *f*_sky_) in the case of a wide-field AtLAST survey (adapted from Raghunathan *et al.*, 2022, which we refer to for details). We include as a reference the residual noise curve expected in the case of the CMB-HD survey ( Sehgal *et al.*, 2019. The shaded band denotes the power spectrum for a fiducial thermal SZ sky as extracted from the BAHAMAS simulations ( McCarthy *et al.*, 2017), with 1 *σ* and 2 *σ* credible intervals based on George *et al.* (2015).

In Figure 10 we present the result of an optimal internal linear combination of simulated multi-frequency AtLAST maps when adopting a wide-field survey strategy over a period of 5 years. The proposed spectral configuration will in particular allow for reaching a lower mass limit almost a factor of 2× lower than achievable with CMB-HD ( Sehgal *et al.*, 2019), a reference next-generation CMB facility in terms of proposed survey depth, and with better angular resolution. This implies that AtLAST will be able to probe the SZ signal to Compton *y* levels ≲ 5×10 ^−7^ over an extreme dynamic range of spatial scales when considering a deep survey approach (< 4000 deg ^2^). Targeted observations will allow us to reach a beam-level Compton *y* depth of ∼ 2×10 ^−6^ per hour of integration time. Previous studies (e.g., Dolag *et al.*, 2016; Raghunathan, 2022) have predicted a Compton *y* confusion floor of 2−5×10 ^−7^ from < 10 ^13^ M _⊙_ haloes (corresponding to predicted detection threshold for AtLAST). As such, the estimated sensitivity imply that AtLAST will obtain SZ confusion limited observations in ∼ 100 h with single-pointing strategy. Nevertheless, we note that this sensitivity estimate corresponds to the residual Compton *y* root-mean-square noise obtained when applying a constrained internal linear combination procedure to a simple set of mock AtLAST observations. In particular, we generate flat-sky sky realizations at the AtLAST bands including Galactic foregrounds and extragalactic background. The foreground model is based on the pysm3 models ( Thorne *et al.*, 2017), but the code was ported and adapted to extend the stochastic components down to arcsecond scales. The output mock realization comprises the dust ( d11 model), AME ( default), free-free ( default) and synchrotron ( s6) from the Milky Way. The background signal is composed by a random CMB realization, as well as infrared and radio background from unresolved sources as extracted from the SIDES ( Béthermin *et al.*, 2017) and RadioWeb-Sky simulations ( Li *et al.*, 2022), respectively. To reproduce a clean subtraction of any dominant contaminating compact sources, we excluded all the radio and infrared components with fluxes in at least two bands larger than 3× the corresponding noise root-mean-square. As such, it should be considered as a rough ground reference for the actual depth achievable with future AtLAST measurements. Future forecasting studies will particularly investigate how different observation strategies, source subtraction, and modeling techniques will affect the contamination mitigation and the effective SZ sensitivity.

**Figure 11.**
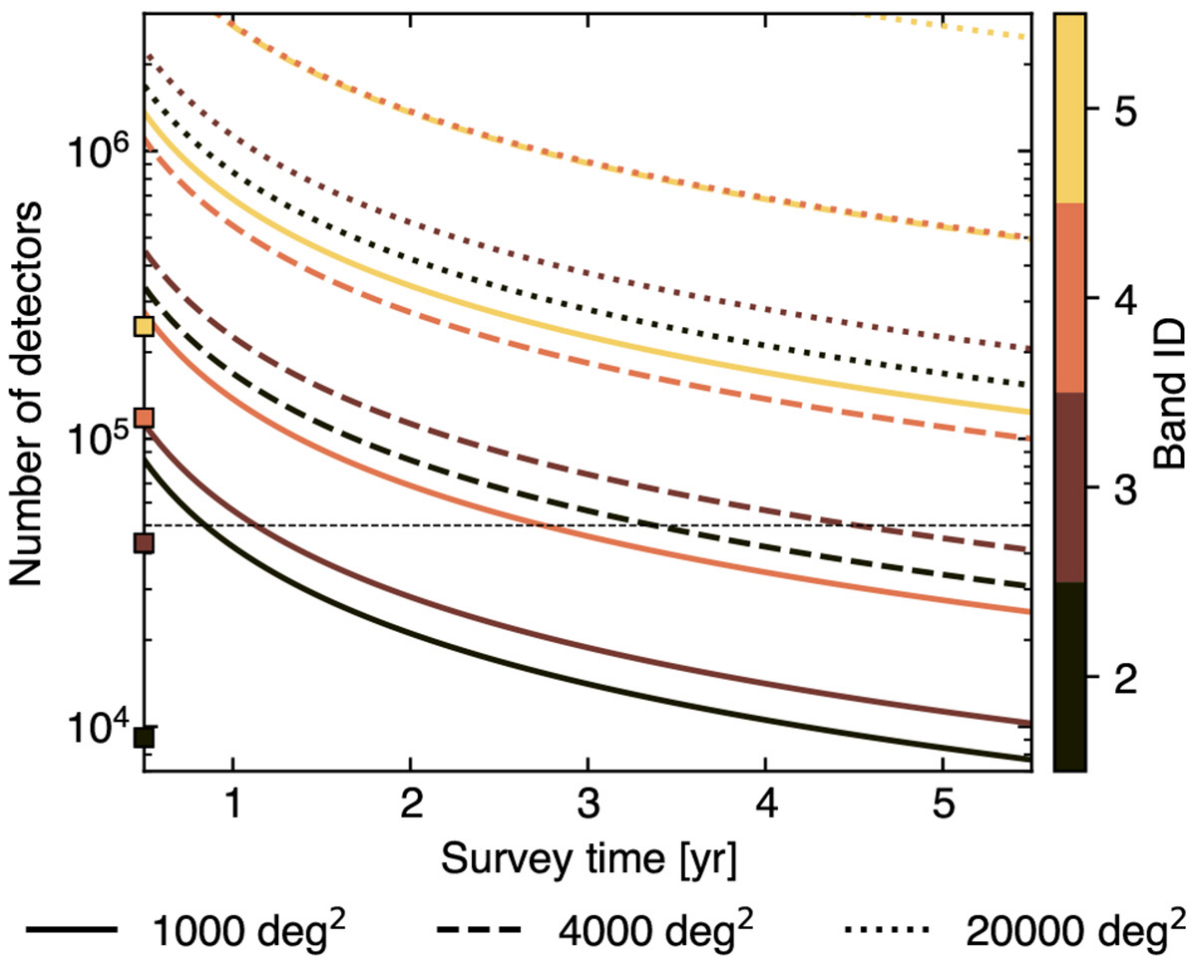
Required number of detectors to reach the target sensitivity estimates listed in Table 1 for different bands and considering different survey strategies. For comparison, the squares on the ordinate marks the detector counts required in each band for fully covering a 1 deg ^2^ field of view. The horizontal line traces the minimal number of detectors (50,000) identified for reaching the target depth in Band 2 and 3 in the case of 1000 deg ^2^ and 4000 deg ^2^ surveys. We note that this is consistent with the estimated specifications reported in the AtLAST Memo 4 for the 1 ^st^ generation instruments.

Still, achieving such frontier capabilities will necessarily demand a considerable mapping speed and, thus, a crucial effort in the optimization of the detector array. To estimate a minimal detector count for filling the focal plane, we consider the sensitivity estimates reported in Table 1 as target depths for surveys with varying observing period and sky coverage (see Figure 5). The results are reported in Figure 11. A detector count *n*_det_ ≃ 5 × 10 ^4^ is sufficient for achieving the sensitivity goal in the case of a narrow survey configuration (1000 deg ^2^) both in Band 2 and Band 3, key for tracing the decrement regime of the thermal SZ signal. For the same bands, the same *n*_det_ constraints would allow to achieve a similar survey sensitivity also in the intermediate 4000 deg ^2^ case over ∼ 4 − 5 years. Nevertheless, extending these considerations to other bands or a wide-field scenario would require a significant increase in *n*_det_. For instance, in the case of Band 5—crucial for constraining the departures from the thermal SZ effect due to kinetic and relativistic contributions — such a boost would range over almost an order of magnitude.

As broadly highlighted in Section 2, constraining the small-scale fluctuations in the thermal and kinetic SZ effects, while tracing the temperature-dependent relativistic SZ corrections would imply measuring deviations from the global SZ distribution order of magnitudes smaller than the bulk, non-relativistic thermal SZ signal. This would in turn require a significant reduction of any systematic effects hampering the overall calibration accuracy. In this regard, an interesting technical aspect of AtLAST is the plan for closed-loop metrology for tracking the alignment of the primary mirror panels (see, e.g., Mroczkowski *et al.*, 2024; Reichert *et al.* in prep.). By using active, closedloop metrology such as the laser system currently being developed for the Sardinia Radio Telescope ( Attoli *et al.*, 2023) or the wavefront sensing system being developed on the Nobeyama Radio Observatory 45-m ( Nakano *et al.*, 2022; Tamura *et al.*, 2020) the errors in the beam can be kept down to sub-percent levels, meaning the beam will be diffraction limited and stable throughout observations. This in turn will improve the calibration accuracy and reduce systematics (see, e.g., Naess *et al.*, 2020 for discussion of the diurnal effects on the ACT beams) that have been shown to impact CMB and SZ results at the several percent level in the case of passive optics (3 − 5%; Hasselfield *et al.*, 2013; Lungu *et al.*, 2022), with the result that the daytime data have generally been excluded from cosmological analyses. Future dedicated forecasts will analyze the benefits of metrology for secondary CMB measurements using AtLAST, including improvements to the calibration, reduction of systematics, and the ability to recover larger angular scales on sky. However, the salient takeaway message is that uncertainties in the beam should no longer be a leading source of systematic error.

### 4.3 Mock reconstruction of the relativistic SZ effect

The relative amplitude of the relativistic component compared to the thermal and kinetic SZ effects makes this modeling task highly challenging. To test the prospects of using AtLAST measurements for performing a spectral separation and analysis of the SZ effect, we thus perform a mock reconstruction of the intracluster temperature using the relativistic SZ effect.

**Figure 12.**
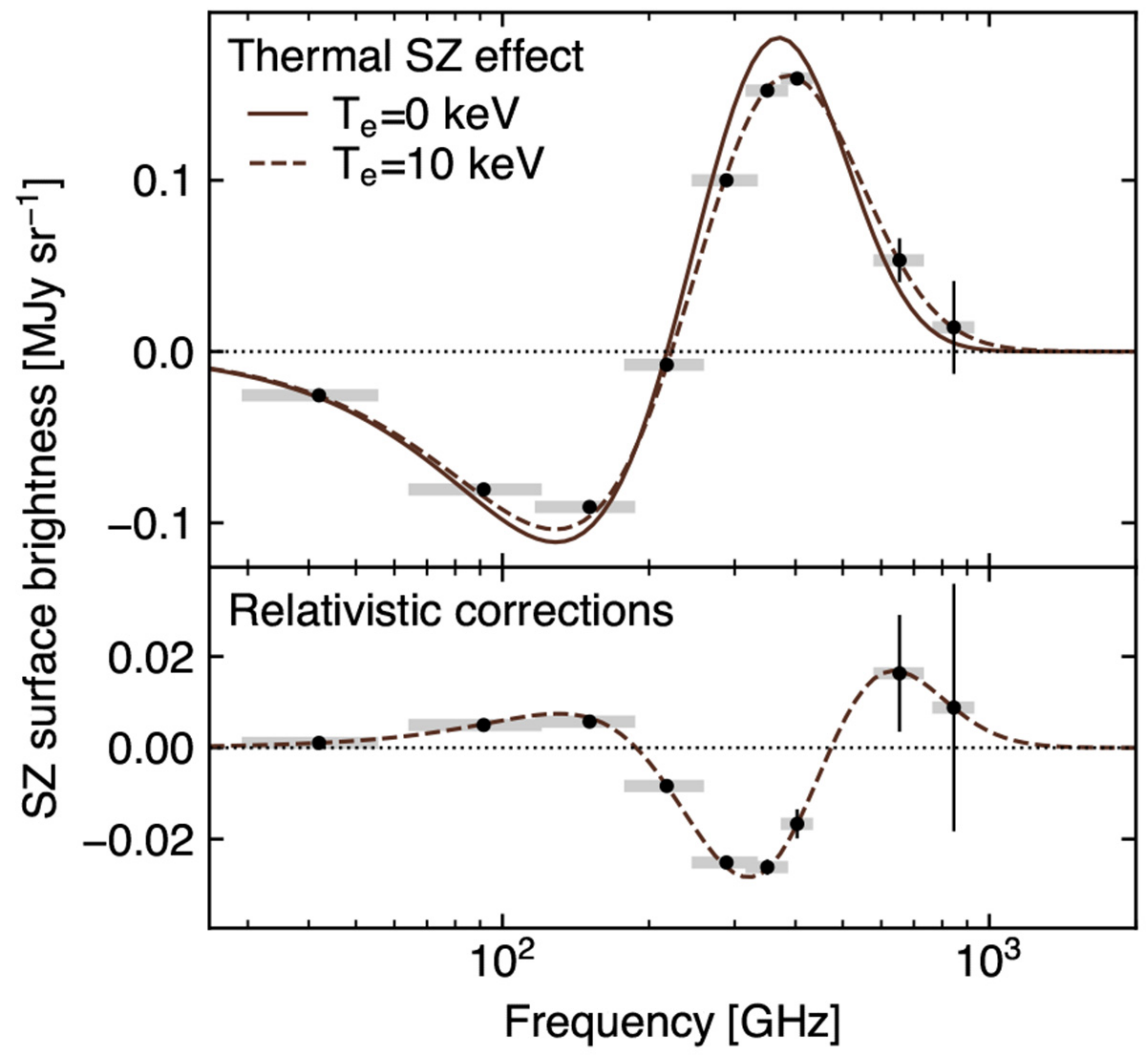
Predicted rtSZ measurements for a cluster with a temperature of 10 keV and Compton *y* of 10 ^−4^, assuming flat bandpasses in the Bands 2–10 (denoted as gray bands; see also Table 1). We assume the same exposure time in each band and account for flux sensitivity and beam size differences, tuned to achieve a SNR = 50 in Band 8. For comparison, the non-relativistic approximation is also shown. The bottom axis shows the difference between the relativistic and non-relativistic spectra.

***4.3.1 SZ-only reconstruction*.** As a test case, we consider a galaxy cluster with temperature *T*_SZ_ = 10 keV and Compton *y* = 10 ^−4^. We note that, despite representing relatively extreme (but realistic) values, the setup ( *T*_SZ_, *y*) = (10 keV, 10 ^−4^) is chosen to facilitate this first study of the AtLAST capabilities of providing spectral constraints on temperature-dependent distortions of the thermal SZ effect. A broader exploration of the parameter space will be presented in Section 4.3.3. The amplitude of the SZ signal at each of the selected bands in the minimal spectral set is obtained by integrating the relativistically-corrected thermal SZ (rtSZ) spectrum across each band assuming flat bandpasses. We then obtained estimates for the corresponding uncertainties based on the sensitivity estimates from the AtLAST sensitivity calculator. First, we compute the integration time required to achieve a signal-to-noise (SNR) of 50 in Band 8, arbitrarily chosen among the two spectral windows closest to the peak in the rtSZ effect ( Figure 2 & Figure 12). The resulting noise root-mean-square (RMS) is defined as the corresponding uncertainty. The uncertainties *δI* for each of the remaining bands are thus computed assuming the same integration time as for the Band 8 estimation above, but taking into account both the differing point-source sensitivities and beam sizes across frequency bands,

$$\frac{\delta I\left(n\right)}{\delta I\left(8\right)}=\frac{\sigma \left(n\right)}{\sigma \left(8\right)}\times \frac{\Omega \left(8\right)}{\Omega \left(n\right)}.\;(1)$$

Here, *n* denotes the band index ( *n* = {2, ..., 10}), while *σ* and Ω( *ν*) are the flux density RMS and the beam size at a given frequency *ν*, respectively. The resulting simulated measurements are shown in Figure 12.

**Figure 13.**
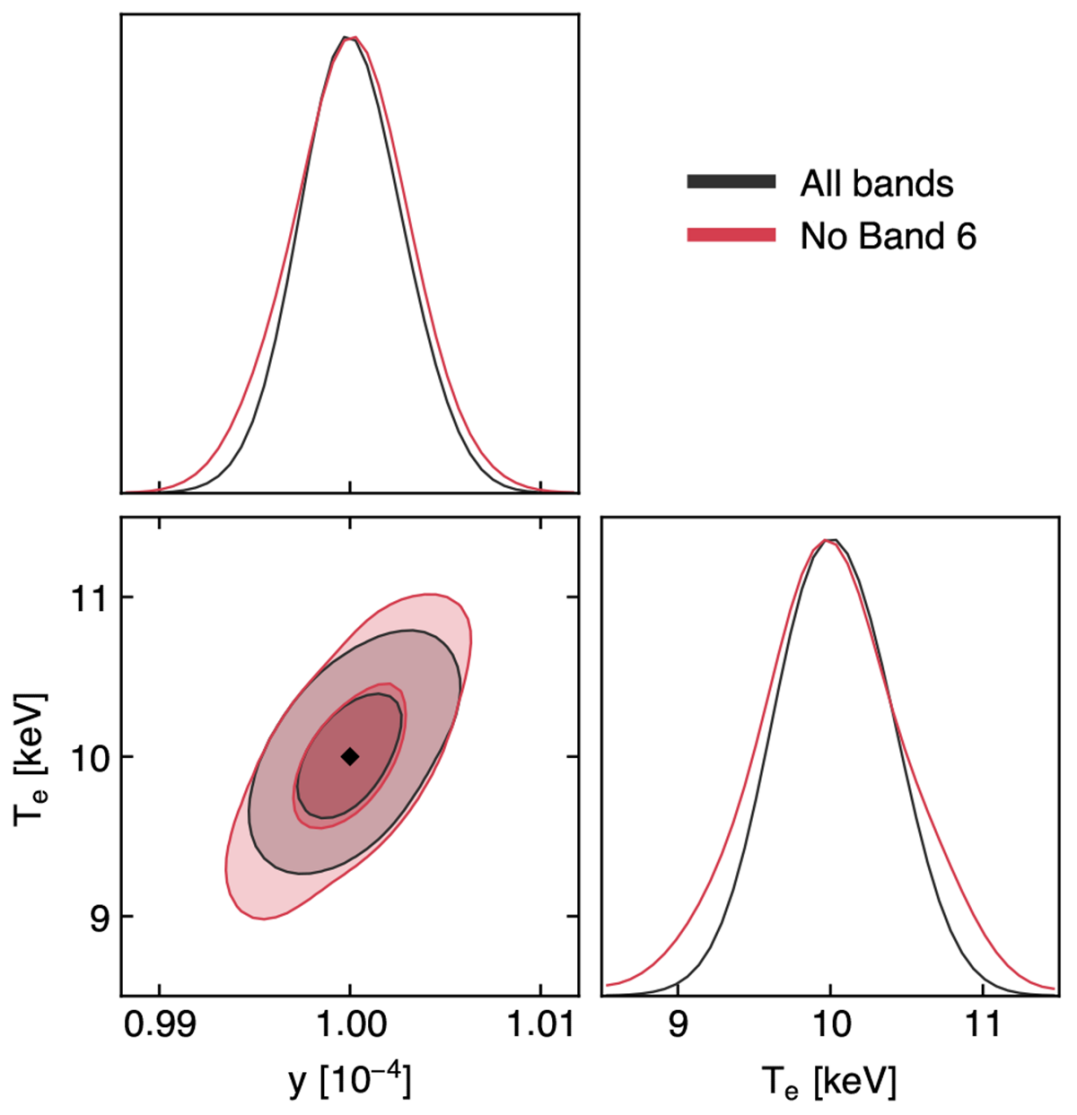
Fits to the simulated measurements shown in Figure 12, using all bands (grey) and all except Band 6 (red). Excluding Band 6 increases the *y*- *T*_e_ degeneracy substantially. The black diamond denotes the input parameters.

The derived SZ measurements can then be used to perform a simple joint inference of the Compton *y* and electron temperature for the target case. If only lower-frequency data points (≲ 200 GHz) are measured, then there is a complete degeneracy between *T*_e_ and the Compton *y* parameter. In this spectral range, in fact, an increase in the electron temperature reduces the signal in a similar manner as decreasing the overall Compton *y* amplitude. When higher-frequency points are included, the degeneracy can be minimized as shown in Figure 13. By dropping one band at a time from the fit, we find that Band 6 (≈ 240 GHz) has the greatest influence in breaking the degeneracy since the signal-to-noise on the difference between nearly-degenerate models is greatest in this band.

**Figure 14.**
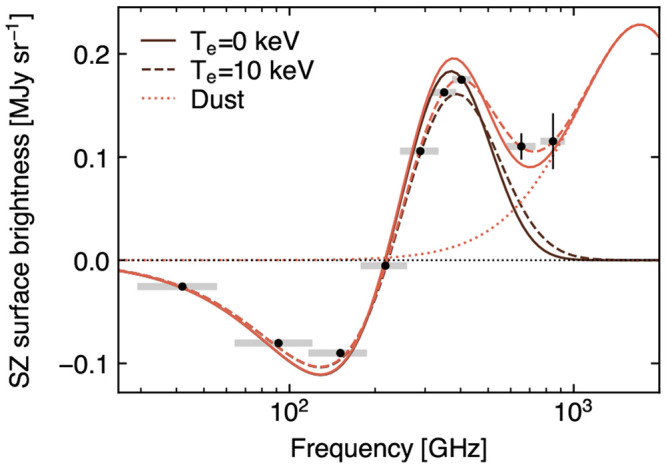
Same as Figure 12, but with the addition of a modified black body dust component based on the model from Erler *et al.* (2018). The red dotted line shows the dust signal. The red solid and dashed lines show the total signal from the dust and non-relativistic and relativistic signals respectively.

***4.3.2 SZ+dust reconstruction*.** So far, we have assumed that the only signal present is the SZ signal. However, in reality there will of course be other astrophysical foregrounds and backgrounds present along the line of sight, resulting in non-negligible contamination of the overall SZ signal observed in the direction of a galaxy cluster. In this test, we however assume that signals that are not spatially correlated to the SZ effect can be removed by means of component separation methods (see Section 4.2) or targeted forward modeling procedures in the case of unresolved compact sources (e.g., Andreon *et al.*, 2021; Di Mascolo *et al.*, 2019a; Kéruzoré *et al.*, 2020; Kitayama *et al.*, 2020; Ruppin *et al.*, 2017). However, previous studies (e.g. Erler *et al.*, 2018) showed that there is a spatially correlated signal within clusters associated with the diffuse dust emission. To understand the impact on the capability of AtLAST in constraining any rtSZ deviation, we repeat the above test by adding an additional dust-like spectral component ( Figure 14). In particular, we assume a modified black body signal given by ( Erler *et al.*, 2018)

$$I_{\text{Dust}}\left(\nu \right)=A_{\text{dust}}^{857}{\left(\frac{\nu }{\nu_{0}}\right)}^{\beta_{\text{Dust}}+3}\frac{\text{exp}\left[h\;\nu_{0}/\left(k_{B}T_{\text{Dust}}\right)\right]-1}{\text{exp}\left[h\;\nu /\left(k_{B}T_{\text{Dust}}\right)\right]-1},\;(2)$$

where *ν*_0_ = 857 GHz is chosen as the reference frequency, and $A_{\text{dust}}^{857}$ is the amplitude at this frequency. We use the ( Erler *et al.*, 2018) parameter fits for $A_{\text{dust}}^{857}$ , *β*_Dust_ and *T*_Dust_ to generate a dust signal, and add them as free parameters to our fit with uniform priors on all parameters. The uncertainties on the measurements in each band are the same as in the SZ-only fit ( Section 4.3.1)

**Figure 15.**
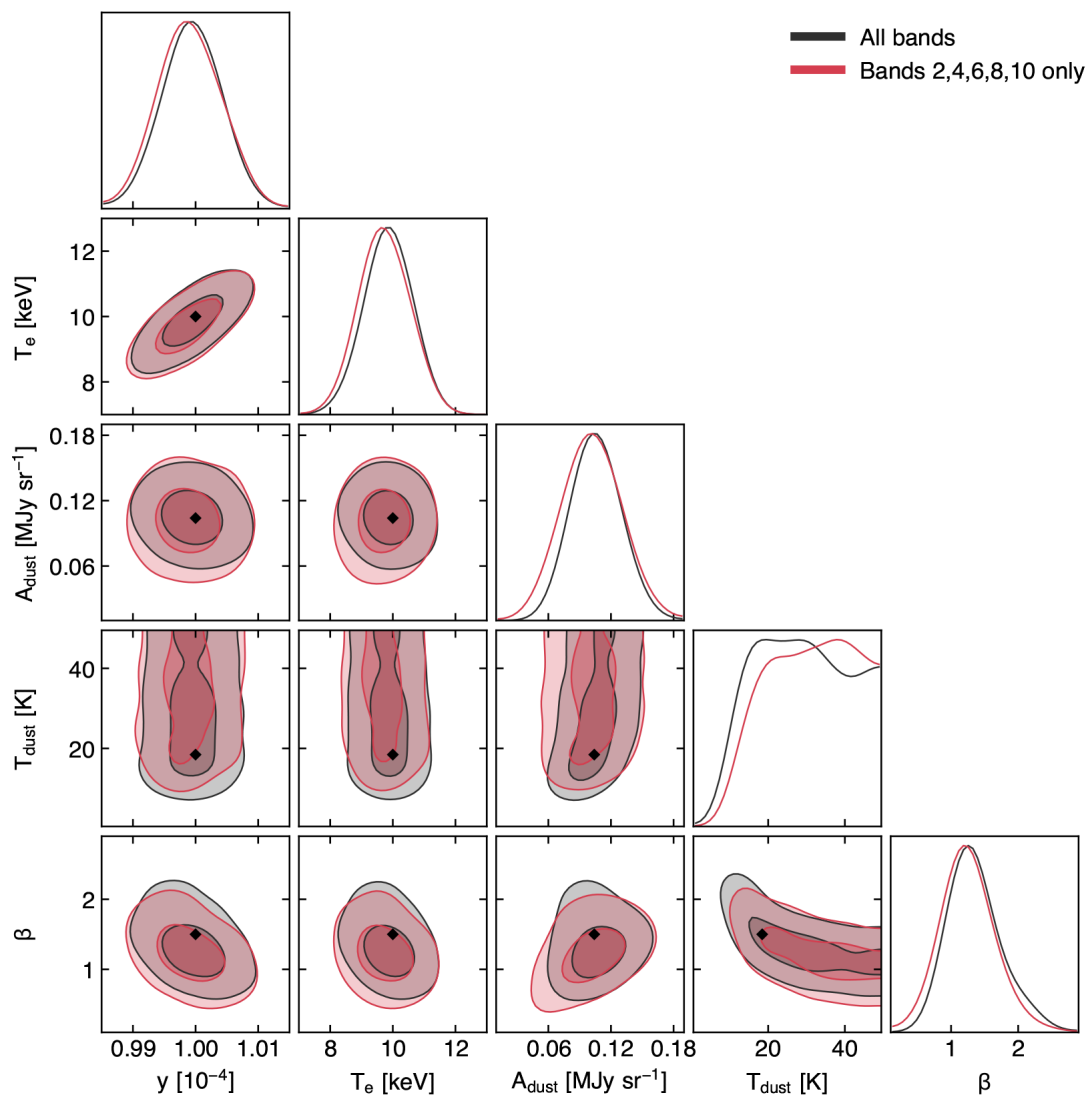
Fits to the simulated measurements shown in Figure 14, using all bands (grey) and Bands 2, 4, 6, 8 and 10 only (blue). With this optimal set of five bands, the constraints on the rtSZ parameters are almost equivalent to the constraints with all bands, but we obtain a slightly reduced constraining power on the dust parameters. The dotted diamonds denote the input parameters.

In this case, more bands become necessary to correctly constrain the rtSZ temperature and disentangle the rtSZ and dust spectral components. The best minimal combination comprises Bands 2, 4, 6, 8 and 10, that provide almost identical constraints to the full set of bands on the rtSZ parameters, while achieving a lower precision on the dust parameters (as shown in Figure 15). Most importantly, it is important to note that the broad spectral coverage offered by the proposed setup allows a clean separation of the rtSZ and dust signals with only a marginal impact on the rtSZ constraints compared to the SZ-only case ( Section 4.3.1).

***4.3.3 Required sensitivity and time forecasts*.** The reference SNR of 50 employed above was mainly intended to achieve a general perspective on the spectral constraining power of the proposed setup without being limited by the inherent significance of the test SZ signal. Thus, we now investigate what SNR is required to achieve good temperature constraints from rtSZ measurements. In particular, we run similar fits for different values of the reference SNR and different temperatures.

**Figure 16.**
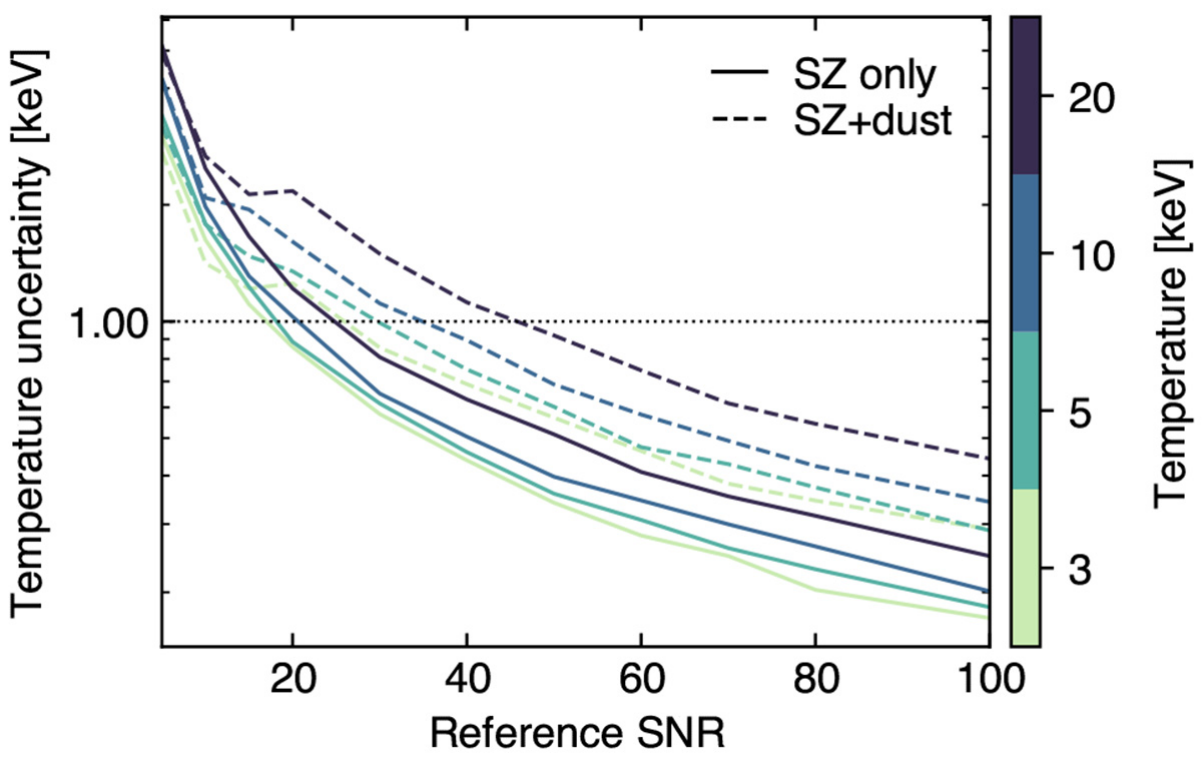
Precision achieved on the temperature reconstruction as a function of the SNR in the reference band (384–422 GHz; Band 8) and for a range of ICM temperatures. The horizontal line denotes the target temperature *T*_SZ_ = 1 keV discussed in the text.

The impact of the varying SNR on the temperature reconstruction is summarized in Figure 16. If we require, for example, an accuracy of 1 keV in temperature, a reference SNR of 40 is sufficient for all the temperatures tested. A reference SNR of 30 is instead sufficient for all except the very hottest clusters when dust is included.

Although AtLAST will be able to observe clusters spanning a broad range in mass and redshift (and, hence, temperature), the example analysis presented in the previous section is aimed only at forecasting AtLAST capabilities of measuring relativistic deviations from the standard thermal SZ and not at testing the expected detection threshold as a function of cluster properties. To take into account the evolution of the rtSZ effect with the mass and redshift of a galaxy cluster, we aim here at estimating the required observing time to reach a target SNR in Band 8, our reference spectral window ( Section 4.3.1).

**Figure 17.**
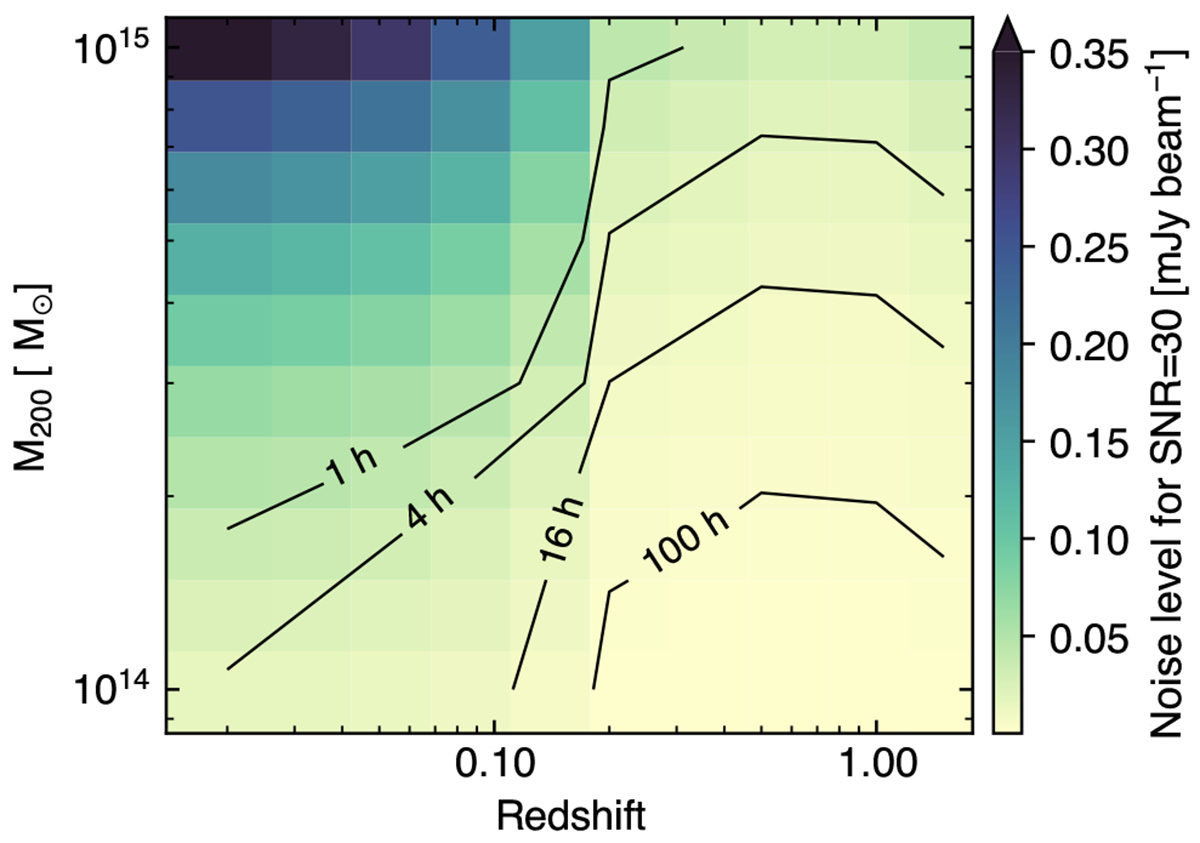
Beam-level noise root-mean-square and observing times as a function of cluster redshift and mass *M*_200_ required to reach an SNR of 30 in the reference spectral band (384–422 GHz; Band 8), allowing average SZ temperatures to be well constrained.

To do so, we construct cluster signal maps for a range of masses and redshifts, using the physical model given in Olamaie *et al.* (2012) and Javid *et al.* (2019). Assuming hydrostatic equilibrium, this model gives us physically consistent pressure and temperature profiles which we use with the szpack ( Chluba *et al.*, 2012; Chluba *et al.*, 2013) temperature-moment method to predict relativistic SZ effect signal maps, taking into account the spatial variation of the temperature. The resulting observing time predictions for a reference SNR of 30 are shown in Figure 17. For reasonable observing times (< 16 hours) we can get average temperature constraints for most clusters at redshifts up to *z* ≈ 0.1, and high-mass clusters ( *M*_200_ ≳ 4 × 10 ^14^M _⊙_) up to arbitrarily high redshift. It is important to note that the enhanced angular resolution of AtLAST could easily allow one to obtain spatially resolved information on the temperature distribution, once the SNR requirements are satisfied for each spatial element considered for the analysis — e.g., radial bins or spectrally homogeneous regions as generally considered in high-resolution X-ray studies (e.g., Sanders, 2006).

## 5 AtLAST SZ studies in a multi-probe context

AtLAST will provide an unprecedented speed and spectral grasp across the (sub)millimeter spectrum. This will make AtLAST inherently relevant beyond just SZ science, and will open up possibilities for fundamental synergies in the a multi-wavelength and multi-probe exploration of the Universe.

### 5.1 AtLAST scientific cross-synergies

Thanks to the novel multi-instrument design ( Mroczkowski *et al.*, 2024), AtLAST will be aimed at representing a high-impact (sub)millimeter facility with a broad and varied scientific reach. As such, this will set the ground for a natural cross-synergy across the different scientific applications identified as part of the AtLAST Science Development effort.

In the case of a wide-field continuum survey discussed Section 4.2, the multi-band coverage and the extended temporal span will make the SZ-driven observations extremely valuable for temporally-dependent studies as for transient surveys (Orlowski-Scherer *et al.* in prep.). Similarly, the multiple bands and likely polarization sensitivity will be useful in the study of Galactic dust and molecular clouds ( Klaassen *et al.*, 2024), building on the lower resolution results with, for example, the Simons Observatory ( Hensley *et al.*, 2022).

Related to the science goals proposed in this work, the availability of a wide-field spectroscopic survey of the distant Universe ( van Kampen *et al.*, 2024) will immediately enhance the validity of the SZ identification and study of high- *z* clusters and protoclusters by providing accurate redshift information. The broad spectral coverage achieved thanks to the proposed multi-band setup will actually play a crucial role in maximizing the redshift domain. At the same time, as already mentioned in previous sections, having access simultaneously to constraints on the physical properties of large-scale environments via the SZ effect ( Section 3.5) and on the associated galaxy populations (via the spectral characterization of their cold molecular gas and the inference of their dust content; see Figure 9) will represent an unprecedented opportunity in the context of galaxy-environment co-evolution studies.

Similarly, the information on the warm/hot component of galactic haloes will be essential for building a comprehensive picture of the diffuse and multi-phase CGM ( Lee *et al.*, 2024). A combination of the novel perspective offered by AtLAST on the cold contribution with the tight measurements of the thermal and kinetic properties of such elusive haloes ( Section 3.6) will represent the only way for shedding light on the many potential evolutionary routes of the elusive large-scale CGM.

### 5.2 Synergies with other state-of-the-art and forthcoming facilities

In the context of large scale structures, AtLAST’s constraints on the multi-faceted SZ effect will be highly complementary to multi-wavelength information on the galaxy motions and distribution, the gravitational potentials of the systems, the X-ray emission, magnetic field structure, and the highly non-thermal and relativistic emission traced by radio emission. Below, we highlight some of the key facilities and experiments that provide the most synergy with AtLAST.

***5.2.1 Radio*.** The radio waveband offers information that can complement and enhance many of the science cases outlined above, and next-generation instruments such as the Square Kilometer Array (SKA; Huynh & Lazio, 2013) and the next-generation Very Large Array (ngVLA; Selina *et al.*, 2018) will have the angular resolution and sensitivity required to provide it. On the one hand, getting a clear view of the resolved SZ effect and searching for intrinsic scatter and surface brightness fluctuations requires sensitive detection and removal of contaminating radio sources (e.g., Dicker *et al.*, 2021). While this will already be possible with AtLAST’s data itself thanks to its spectral coverage and ≈ 5″ *λ* mm ^−1^ resolution (i.e. 10″ at 2 mm), interferometric observations at lower frequency (SKA-MID) will aid in pinpointing the location and morphology of the radio sources, while also being more sensitive to fainter sources as most will be brighter at lower frequency.

On the other hand, radio information provides a powerful complementary probe of the astrophysics in the cluster, being sensitive to magnetic fields and populations of non-thermal electrons. The reference surveys proposed for the SKA ( Prandoni & Seymour, 2015) predict the detection of ∼1000s of radio halos with SKA1-LOW, out to redshifts of at least 0.6 and masses *M*_500_ > 10 ^14^ M _⊙_ ( Cassano *et al.*, 2015; Ferrari *et al.*, 2015; see also, e.g., Knowles *et al.*, 2021, Knowles *et al.*, 2022, Duchesne *et al.*, 2024 for preliminary results from SKA precursors), along with potential first detections of the polarization of radio halos ( Govoni *et al.*, 2015). This offers the opportunity not only to compare the detailed astrophysics of the thermal and non-thermal components of clusters, shedding light on the turbulent properties currently limiting the accuracy of mass estimation (see Section 3.1), but also to potentially discover new populations of clusters via their radio signals. When also observed by AtLAST, these populations will offer insight into the variation in cluster properties when selecting by different methods (see also Section 3.4). Faraday Rotation Measure observations of polarized sources behind galaxy clusters as well as studies of tailed radio galaxies *in* clusters will enable the study of cluster magnetic fields in unprecedented detail ( Bonafede *et al.*, 2015; Johnston-Hollitt *et al.*, 2015a; Johnston-Hollitt *et al.*, 2015b), contributing to our astrophysical understanding and ability to make the realistic simulations crucial for interpreting observations.

Ultimately, the direct correlation of the SZ information with the spatial, spectral, and polarimetric properties of the multitude of radio structures observed in the direction of galaxy clusters will be essential for constraining the detailed mechanisms governing particle (re)acceleration within the ICM ( van Weeren *et al.*, 2019). Specifically, there is mounting evidence that the non-thermal plasma observed in the form of (multi-scale) radio halos (e.g., Cuciti *et al.*, 2022; Gitti *et al.*, 2015) as well as intercluster bridges (e.g., Bonafede *et al.*, 2022; Botteon *et al.*, 2020b; Radiconi *et al.*, 2022) originates due to turbulent (re)acceleration ( Brunetti & Lazarian, 2007; Brunetti & Jones, 2014; Cassano *et al.*, 2023; Eckert *et al.*, 2017). On the other hand, radio relics are connected to (re)acceleration at shock fronts ( Akamatsu & Kawahara, 2013; Botteon *et al.*, 2020a; van Weeren *et al.*, 2017). While it is clear that cluster mergers are driving both processes (turbulence and shocks), our understanding of the physics of (re)acceleration in clusters is limited by two factors: (i) information about the distribution of gas motions in the ICM is currently sparse and usually inferred via indirect methods (for a review, see Simionescu *et al.*, 2019), and (ii) characterizing shocks in the low-density cluster outskirts, where radio relics are usually found, is very challenging. Detailed mapping of the thermal (sensitive to shocks) and kinetic (sensitive to gas motions) SZ signals throughout the volume of a large sample of galaxy clusters (potentially extending out into the cosmic web), and how these signals relate to features observed in the radio band, will be invaluable towards painting a clear picture of the connection between large-scale structure assembly, magnetic field amplification, and cosmic ray acceleration.

Understanding the impact of AGN feedback, on the other hand, ( Section 3.6) requires complementary observations of the AGN themselves. Gitti *et al.* (2015) finds that even with early SKA1 (50% sensitivity), all AGN with luminosity > 10 ^23^ W Hz ^−1^ can be detected up to *z* ≤ 1 with subarcsecond resolution, and the radio lobes thought to be responsible for carving out the X-ray cavities should be detectable in any medium – large mass cluster at any redshift in the SKA1-MID deep tier surveys. Moreover, SKA1-MID is predicted to detect intercluster filaments at around 2.5 – 6 *σ* ( Giovannini *et al.*, 2015), providing information on their magnetic fields as a complement to the SZ information on their thermodynamic properties ( Section 3.7).

At the top of the SKA frequency range, it will be possible to directly access thermal SZ information. Future extensions to the SKA-MID Phase 1 setup (with the integration of the high-frequency Band 6; we refer to the SKA Memo 20-01 for details) and the ones envisioned for SKA Phase 2 (2030+) will allow SKA to probe the low-frequency (≲ 24 GHz) domain of the SZ spectrum, less affected by kinetic and relativistic deviations than the range probed by AtLAST. In fact, Grainge *et al.* (2015) find that 1 hour of integration is sufficient for obtaining a 14 *σ* detection of the SZ effect from a *M*_200_ = 4 × 10 ^14^ M _⊙_ cluster at *z* > 1. On the other hand, the clean perspective offered by AtLAST on the multiple SZ components will provide the means, e.g., for cleanly disentangling the SZ footprint of galaxy clusters from the faint, diffuse signal from mini- to cluster-scale radio haloes, large-scale relics, and back-/foreground and intracluster radio galaxies, enhancing their joint study.

***5.2.2 Millimeter/submillimeter*.** Millimetric/submillimetric survey experiments like SO ( Simons Observatory Collaboration, 2019) and its upgrades, CCAT-prime/FYST ( CCAT-Prime Collaboration, 2023), upgrades to the South Pole Telescope ( Anderson *et al.*, 2022), and ultimately CMB-S4 ( Abazajian *et al.*, 2016) will cover roughly half the sky over the next few years to a decade, predominantly in the Southern sky. Along with past and current facilities, these however have ∼ arcmin resolution, well-matched to the typical angular size of clusters in order to optimize their detection but not optimal for peering inside clusters to explore astrophysical effects (aside from few nearby exceptional clusters). Nevertheless, while limited to resolutions approximately 8.33× lower than AtLAST at the same frequencies, their data will provide robust constraints at large scales, lending itself naturally to joint map-making and data combination, as well as valuable source finders for deep AtLAST follow-up. On the other hand, AtLAST will be able to resolve any structures probed by these wide-field surveys, defining a natural and intrinsic synergy.

At still higher resolutions, ALMA will undergo a number of upgrades improving its bandwidth and sensitivity over the next decade. These upgrades are called the Wideband Sensitivity Upgrade (WSU; Carpenter *et al.*, 2023), which in the context of SZ science could deliver 2 − 4× ALMA’s current bandwidth. Wide field mapping capabilities are however not part of the key goals for the WSU, and it is unlikely ALMA will ever map more than a few tens of square arcminutes. Nevertheless, the improved sensitivity and bandwidth could allow for exploiting ALMA to complement AtLAST observations with a high spatial resolution view of astrophysics through detailed follow-up studies. At the same time, such observations will require AtLAST to recover more extended scales (see Section 4). The necessity of such a combination will however allow us to fully leverage the synergistic strengths of single-dish and interferometric facilities for gaining an unprecedented view of the hot baryonic content of the Universe, along with its multi-phase counterparts.

***5.2.3 Optical/infrared*.** The *Euclid* mission ( Euclid Collaboration *et al.*, 2022; Laureijs *et al.*, 2011) has recently started surveying the optical/infrared sky, and is expected to result in the identification of ≳ 10 ^5^ galaxy clusters and protoclusters across the entire cluster era (0 ≲ *z* ≲ 2; Euclid Collaboration, 2019). Complemented with data from the Legacy Survey of Space and Time (LSST) survey by the forthcoming Vera C. Rubin Observatory ( Ivezić *et al.*, 2019), these will represent a wealth of complementary constraints on the cluster and protocluster populations that will be essential for enhancing the scientific throughput of AtLAST in the context of SZ studies. The characterization of the weak lensing footprint of galaxy clusters and groups jointly with resolved information on the thermodynamics of their ICM will enable a thorough exploration of the many processes biasing our cluster mass estimates ( Section 3.1). At the same time, the detailed characterization of the SZ signal from the vast number of weak-lensing selected systems (and, thus, with different selection effects than surveys relying on ICM properties) will allow for studying in detail the origin of the under-luminous clusters ( Section 3.4). Similarly, AtLAST will provide an unprecedented view on the ICM forming within the wealth of high- *z* galaxy overdensities that will be identified by *Euclid*/LSST, in turn providing an unbiased means for constraining the physical processes driving the thermalization of protoclusters complexes into the massive clusters we observe at *z* ≲ 2.

More in general, the access to a rich set of imaging and spectroscopic measurements by wide-field surveys — *Euclid*, Rubin Observatory, and the next generation Nancy Grace Roman Space Telescope ( Spergel *et al.*, 2015), SPHEREx ( Doré *et al.*, 2014) — along with deep, targeted observation from high-resolution facilities — e.g., JWST ( Gardner *et al.*, 2006), or the upcoming Extremely Large Telescope — will be greatly complemented by the resolved, wide perspective of AtLAST on the SZ Universe. Tracing the faint warm/hot backbone of large-scale structure ( Section 3.7), as well as tightly correlating resolved thermodynamic constraints for the large-scale cluster environment with the physical properties of the galaxies embedded within them ( Alberts & Noble, 2022; Boselli *et al.*, 2022) and the distribution of the more elusive intracluster light ( Contini *et al.*, 2014), will be essential to shed light on their complex and dynamical co-evolution.

In addition, to facilitate precision cosmology studies with *Euclid*/LSST, it is imperative to gain a better understanding of the impact of galactic processes on the redistribution of baryons over large scales. Different prescriptions of feedback employed in various cosmological simulations alter the predicted amplitude and scale dependence of the matter power spectra at separations under 10 Mpc on a level that is considerably larger than the statistical uncertainty expected from upcoming cosmology experiments ( Chisari *et al.*, 2019; van Daalen *et al.*, 2020). Mapping the gaseous contents in the low-density outskirts of galaxy groups and WHIM filaments through sensitive AtLAST measurements will thus provide invaluable observational priors necessary to model baryonic feedback for survey cosmology.

***5.2.4 X-ray*.** On the X-ray side, *Chandra* and *XMM-Newton*, launched in 1999 with CCDs capable of 0.5 − 5 *″* spatial resolution, are still providing a reasonable thermodynamic mapping of the brightest regions of the collapsed structures up to redshift ∼ 1.2. The *eROSITA* telescope (launched in 2019) has recently delivered the first release of its X-ray all-sky surveys ( Merloni *et al.*, 2024) and new catalogs of cluster candidates up to redshift *z ≃* 1.3 ( Bulbul *et al.*, 2024). Still, the large point spread function (∼ 15 *″*) and the limited sensitivity does not allow for resolving the temperature structure of the ICM and any derived quantities (e.g., pressure, entropy, mass) with the exception of nearby and bright galaxy clusters (e.g., Iljenkarevic *et al.*, 2022; Liu *et al.*, 2023; Sanders *et al.*, 2022; Whelan *et al.*, 2022).

The 5 eV spectral resolution of the *Resolve* microcalorimeter onboard *XRISM*, launched in September 2023, will soon enable the first systematic investigation of the gas kinematics in hot, X-ray bright galaxy clusters. However, these studies will be limited by the low (∼ 1.3′) angular resolution, small field of view and effective area, especially at soft X-ray energies. This particularly hinders the study of less massive haloes (galaxy groups and CGM) and the mapping of extended cluster outskirts and WHIM filaments. Forthcoming space missions are expected to improve all these performances through the development of next-generation instruments with higher both spectral and spatial resolutions over a wider field of view and with a larger collecting area: *Athena* (expected to be adopted as a L-mission by ESA in 2027 for a launch in 2037) will outperform the current satellites thanks to the larger effective area by an order of magnitude with a spatial resolution better than 10 arcsecs; *LEM* (a proposed US Probe mission; Patnaude *et al.*, 2023) is designed to effectively map the thermodynamics and kinematics of the low-density CGM and WHIM using spectral imaging of soft X-ray line emission; *AXIS* (another US Probe proposal) will extend and enhance the science of sensitive, high angular resolution X-ray imaging.

The complementarity of SZ and X-ray measurements of the warm/hot content of cosmic large-scale structures has long represented a valuable asset for a cross-enhancement of the respective astrophysical information. The different dependence of these tracers on the physical properties of the ionized gas has been broadly exploited — from, e.g. obtaining tighter constraints on the thermodynamics of the hot gas in distant clusters and cluster outskirts (e.g., Andreon *et al.*, 2021; Castagna & Andreon, 2020; Ghirardini *et al.*, 2019; Ghirardini *et al.*, 2021b; Lepore *et al.*, 2024; Ruppin *et al.*, 2021) and large-scale filaments (e.g., Akamatsu *et al.*, 2017; Hincks *et al.*, 2022; Planck Collaboration, 2013), to studying local deviations from particle and thermal equilibrium (e.g., Basu *et al.*, 2016; Di Mascolo *et al.*, 2019b; Sayers *et al.*, 2021), deriving detailed morphological models of the three-dimensional distribution of ionized gas (e.g., De Filippis *et al.*, 2005; Kim *et al.*, 2023; Limousin *et al.*, 2013; Sereno *et al.*, 2018; Umetsu *et al.*, 2015), or obtaining measurements of the Hubble–Lemaître parameter independently of more standard probes (e.g., Bonamente *et al.*, 2006; Kozmanyan *et al.*, 2019; Wan *et al.*, 2021).

The enhanced sensitivity, spatial resolution, and mapping speed of AtLAST for various flavors of the SZ effect, combined with the capabilities of next-generation X-ray facilities, will undoubtedly take these already existing synergies one leap further. On the other hand, the novel high spectral resolution imaging capabilities in the soft X-ray band (expected to become available in the next decade with, e.g., *LEM* and *Athena*) will give rise to new opportunities for complementary measurements with the SZ band. Namely, X-ray observations are primarily expected to map the line emission or absorption signals from *metals* in the diffuse, warm-hot gas permeating large-scale structure WHIM filaments or the low-mass haloes of individual L* galaxies. The X-ray continuum emission from these targets will be swamped by the foreground continuum from our own Milky Way, and extremely difficult to probe (see, for instance, Kraft *et al.*, 2022). The ideal path to obtaining a full picture of the physical properties of this diffuse gas component of the cosmic web, therefore, is to combine diagnostics about the metal content (from X-ray line intensities), metal dynamics (from X-ray line widths and shifts), temperature (from X-ray line ratios and relativistic SZ terms) with the gas pressure cleanly measured through the thermal SZ signal. We can then solve for the gas density (knowing the pressure and temperature), and the gas metallicity (knowing the metal content and gas density). Taking this one step even further, by detecting the kinetic SZ signal from the same gas, it will be possible to compare the velocities of metal-poor (primordial) gas from the kinetic SZ measurements which may be different than the velocities of metals probed from the X-ray lines. This will provide truly groundbreaking information about the circulation of gas and metals in and out of galaxies, by offering the opportunity to map, for instance, metal-rich outflows driven by feedback, and metal-poor inflows driven by accretion from the cosmic web, leading to a revolution in our understanding of galaxy evolution.

## 6 Summary and conclusions

AtLAST will provide a transformational perspective on the SZ effect from the warm/hot gas in the Universe. The high angular resolution enabled by the 50-meter aperture, the extensive spectral coverage, and the extreme sensitivity swiftly achievable over wide areas of the (sub)millimeter sky will provide the unprecedented opportunity to measure the SZ signal over an instantaneous high dynamic range of spatial scales (from few arcsecond to degree scales) and with an enhanced sensitivity (≲ 5 × 10 ^−7^ Compton *y*).

Such a combination of technical advances will allow us to constrain simultaneously the thermal, kinematic, and relativistic contribution to the SZ effect for a vast number of individual systems, ultimately opening a novel perspective on the evolution and thermodynamics of cosmic structures. Such an unmatched capability will provide the means for exploring key astrophysical issues in the context of cluster and galaxy evolution.

- By resolving the multi-faceted SZ footprint of galaxy clusters, low-mass groups, and protoclusters, it will be possible to trace the temporal evolution of their thermodynamic properties across (and beyond) the entire cluster era ( *z* ≲ 2), over an unprecedented range in mass. The complementary information on the full spectrum of small-scale ICM perturbations that will be accessed thanks to AtLAST’s superior resolution and sensitivity will thus allow us to build a complete picture of the many intertwined processes that make galaxy clusters deviate from the otherwise hydrostatic equilibrium and self-similar evolution. At the same time, we will be able to get a complete census of the cluster population, circumventing the inherent biases associated with current cluster selection strategies. Overall, such studies will allow AtLAST to be pivotal in firming the role of galaxy clusters as key cosmological probes.
- The possibility offered by AtLAST of accessing the low-surface brightness regime will open an SZ window on the low-density warm/hot gas within the cosmic large-scale structure — ranging from the characterization of the mostly unexplored properties of the assembling ICM seeds within protocluster overdensities to the barely bound outskirts of galaxy clusters. These represent the environments where the same process of virialization begins. As such, they are ideal for studying how deviations from thermalization, gas accretion, and strong dynamical processes impact the thermal history of galaxy clusters.
- By tracing the imprint on the thermodynamics properties of circumgalactic medium surrounding galaxies and of the cluster cores, AtLAST will allow to constraint energetics and physical details of AGN feedback. This will provide the means for moving a fundamental step forward in our understanding of the crucial impact of AGN on the evolution of the warm/hot component of cosmic structures over a wide range of spatial scales and across cosmic history.

To achieve these ambitious goals, it will be essential to satisfy the following technical requirements:

- **Degree-scale field of view.** The superior angular resolution achievable thanks to the 50-meter aperture planned for AtLAST will need to be complemented by the capability of effectively recovering degree-level large scales. Such a requirement is motivated by the aim of mapping the SZ signal from at low or intermediate redshift astrophysical sources that are inherently extended on large scales (e.g., intercluster filaments) and with diffuse signals (e.g., protocluster overdensities). At the same time, we aim at performing a deep (∼ 10 ^−7^ Compton *y*) and wide-field (> 1000 deg ^2^) SZ survey, key for effectively probing a varied sample of SZ sources. In turn, our requirement consists of an instantaneous field of view covering > 1 deg ^2^. Clearly, combining wide-field capabilities with enhanced sensitivity will be highly demanding in terms of minimal detector counts. To reach the target sensitivities reported in Table 1, we forecast that the focal plane array should be filled by ≳ 50, 000 detectors.
- **Wide frequency coverage.** To perform a spectral inference of the multiple SZ components, along with their clean separation from foreground and background astrophysical contamination, it will be crucial to probe the spectral regime from 30 GHz up to 905 GHz with multi-band continuum observations. We specifically identify an overall set of nine spectral bands (centered at 42.0, 91.5, 151.0, 217.5, 288.5, 350.0, 403.0, 654.0, and 845.5 GHz), specifically selected to maximize the in-band sensitivity at fixed integration time. By testing this spectral configuration in the context of a mock spectral component separation, we demonstrated that such a choice allows for achieving a clean separation of multiple SZ components, as well as of the signal from dominant contamination sources.
- **Sub-percent beam accuracy.** An accurate calibration will be essential for reducing potential systematics in the small-amplitude fluctuations of the SZ signal associated with local pressure and velocity perturbations, or to relativistic distortions. As such, we require a sub-percent level control of the beam stability.

## Ethics and consent

Ethical approval and consent were not required.

## Data Availability

No data are associated with this article.

## Software availability

The calculations used to derive integration times for this paper were done using the AtLAST sensitivity calculator ( Klaassen, 2024), a deliverable of Horizon 2020 research project “Towards AtLAST”, and available from this link.
