# Supplementary material for: Atacama Large Aperture Submillimeter Telescope (AtLAST) science: Resolving the hot and ionized Universe through the Sunyaev-Zeldovich effect
Source: Open Res Eur. 2025 Jun 5;4:113. Originally published 2024 Jun 10. [Version 2] doi: 10.12688/openreseurope.17449.2 (PMC11950722; doi:10.12688/openreseurope.17449.2)
Supplement: Peer review history

# Reviewer report: Reviewer response for version 2

**DOI:** 10.21956/openreseurope.22160.r55133

**Published:** 2025-06-25

**Authors:** Laura Salvati

**Affiliations:** 1 IAS, Université Paris-Saclay, CNRS, Orsay, France

The authors have answered to and implemented all the suggested modifications, and also have substantially improved the introductory sections, helping the readability of the paper. I believe the paper is ready for indexing.

Is the case presented with sufficient detail to be useful for teaching or other practitioners?

Partly

Is the work clearly and accurately presented and does it cite the current literature?

Partly

If applicable, is the statistical analysis and its interpretation appropriate?

Yes

Are all the source data underlying the results available to ensure full reproducibility?

Not applicable

Are the conclusions drawn adequately supported by the results?

Yes

Is the background of the case’s history and progression described in sufficient detail?

Yes

Reviewer Expertise:

Cosmology and Astrophysics

I confirm that I have read this submission and believe that I have an appropriate level of expertise to confirm that it is of an acceptable scientific standard.

# Reviewer report: Reviewer response for version 2

**DOI:** 10.21956/openreseurope.22160.r55131

**Published:** 2025-06-14

**Authors:** Elia Stefano Battistelli

**Affiliations:** 1 Sapienza University of Rome, Rome, Italy

I recommend this manuscript be approved.

Is the case presented with sufficient detail to be useful for teaching or other practitioners?

Yes

Is the work clearly and accurately presented and does it cite the current literature?

Yes

If applicable, is the statistical analysis and its interpretation appropriate?

Yes

Are all the source data underlying the results available to ensure full reproducibility?

Yes

Are the conclusions drawn adequately supported by the results?

Yes

Is the background of the case’s history and progression described in sufficient detail?

Yes

Reviewer Expertise:

CMB, Sunyaev Zeldovich effect, millimetric instrumentation

I confirm that I have read this submission and believe that I have an appropriate level of expertise to confirm that it is of an acceptable scientific standard.

# Reviewer report: Reviewer response for version 2

**DOI:** 10.21956/openreseurope.22160.r55132

**Published:** 2025-06-14

**Authors:** François-Xavier Désert

**Affiliations:** 1 Institut de Planétologie et d'Astrophysique de Grenoble, Univ. Grenoble Alpes, CNRS, IPAG, Grenoble, France

I would like to thank the authors for taking my comments into account. The sensitivity in both instantaneous and survey modes seem very optimistic.

 The prospect of a large single-dish (sub)millimetre telescope at a high altitude within the next decade justifies the indexing of this comprehensive paper on the potential of SZ science for astrophysics and cosmology.

Is the case presented with sufficient detail to be useful for teaching or other practitioners?

Yes

Is the work clearly and accurately presented and does it cite the current literature?

Yes

If applicable, is the statistical analysis and its interpretation appropriate?

Yes

Are all the source data underlying the results available to ensure full reproducibility?

Yes

Are the conclusions drawn adequately supported by the results?

Partly

Is the background of the case’s history and progression described in sufficient detail?

Yes

Reviewer Expertise:

Clusters of galaxies, CMB, ISM

I confirm that I have read this submission and believe that I have an appropriate level of expertise to confirm that it is of an acceptable scientific standard.

# Reviewer report: Reviewer response for version 2

**DOI:** 10.21956/openreseurope.22160.r55130

**Published:** 2025-06-14

**Authors:** Mathieu Remazeilles

**Affiliations:** 1 Instituto de Física de Cantabria (CSIC - UC), Santander, Spain

The authors have satisfactorily addressed all the concerns I raised in my previous review. As a result, I have updated my approval status accordingly.

Is the case presented with sufficient detail to be useful for teaching or other practitioners?

Yes

Is the work clearly and accurately presented and does it cite the current literature?

Yes

If applicable, is the statistical analysis and its interpretation appropriate?

Yes

Are all the source data underlying the results available to ensure full reproducibility?

Not applicable

Are the conclusions drawn adequately supported by the results?

Partly

Is the background of the case’s history and progression described in sufficient detail?

Yes

Reviewer Expertise:

Cosmology, CMB, foregrounds, component separation

I confirm that I have read this submission and believe that I have an appropriate level of expertise to confirm that it is of an acceptable scientific standard.

# Reviewer report: Reviewer response for version 1

**DOI:** 10.21956/openreseurope.18856.r51896

**Published:** 2025-04-03

**Authors:** Mathieu Remazeilles

**Affiliations:** 1 Instituto de Física de Cantabria (CSIC - UC), Santander, Spain

The paper presents a compelling case for the Atacama Large Aperture Submillimeter Telescope (AtLAST) as a next-generation facility for Sunyaev-Zeldovich (SZ) effect studies. It effectively outlines the scientific motivations, observational requirements, and technical advancements necessary to explore the complex thermal evolution of cosmic structures, from galaxies to clusters and filaments. The authors provide theoretical forecasts to justify AtLAST's remarkable capabilities and its expected contributions to astrophysics.

 The paper is well-structured, comprehensive, and presents a strong scientific rationale for AtLAST. The writing is generally clear and well-supported by references to prior work. However, there are several areas where the manuscript could be improved, particularly in terms of clarity and technical rigour.

 This paper deserves indexing after the authors address the comments below. 

**Main comments:**

 1. While the manuscript discusses the limitations of current and future submillimeter facilities, a more quantitative comparison with upcoming telescopes such as FYST, CMB-S4, and CMB-HD would strengthen the argument. A graphical representation comparing AtLAST's expected frequency coverage, sensitivity, resolution, and field of view with these facilities would help clarify its unique capabilities and how it complements or surpass other projects.

 2. Section 3.3 emphasizes the potential of AtLAST for kinetic SZ (kSZ) studies. However, given the inherent spectral degeneracy between the kSZ effect and CMB temperature anisotropies, how does AtLAST intend to disentangle these signals within the ICM? I would expect AtLAST's high spatial resolution to play a crucial role in overcoming this degeneracy, but this advantage is not emphasized in the discussion of the kSZ science case.

 3. In Figure 3, the Compton-y profiles evolve with redshift. The reason for this evolution should be explicitly discussed, as the thermal SZ effect is generally considered redshift-independent. How much of this evolution is driven by intrinsic astrophysical effects (e.g., AGN feedback, accretion, non-thermal pressure) versus observational biases (e.g., resolution limits, selection effects)?

 4. Despite AtLAST's high instrumental sensitivity, it remains unclear whether the residual foreground contamination can be reduced below the thermal SZ confusion noise or if a 'foreground floor' will persist, preventing accurate separation of faint SZ signals(y = 10^-7) in the WHIM or CGM.

 In particular, Figure 10 shows very low residual foreground contamination from the ILC method compared to the tSZ power spectrum across multipoles. However, how do these residuals compare to the tSZ confusion noise and other SZ-related signals, such as kSZ, relativistic SZ corrections, or non-thermal SZ, which AtLAST also aims to extract? If possible, over plotting these additional spectra in Figure 10 would provide a clearer understanding of their relative importance.

 5. The low residual foreground levels in Figure 10 suggest that the ILC method is highly effective. However, it is unclear whether this is primarily due to the availability of nine sensitive frequency channels by AtLAST or if it results from simplified assumptions in the foreground simulations. Providing more details on the sky simulations used in Section 4.2 and Figure 10 would help clarify this. Specifically:

 Are extragalactic foregrounds (e.g., CIB and radio sources) spatially correlated with the thermal SZ signal in these simulations?

 Are the SEDs of extragalactic foregrounds redshift-dependent?

 6. The terminology “kinetic SZ spectral distortion” in Section 3.3 is misleading, as the kSZ effect does not introduce a spectral distortion but instead follows the same spectral shape as the CMB. Similarly, the middle panel of Figure 4 labels the "Kinetic SZ Compton y parameter", which is confusing because the kSZ effect is not characterized by a y-distortion. Could the authors clarify whether there is a unit conversion applied to represent kSZ in this way?

 7. While beam chromaticity across different frequency bands can be mitigated through deconvolution and reconvolution, intra-band beam variations could introduce spectral distortions and biases in SZ reconstruction, particularly for relativistic SZ and diffuse WHIM studies. Has the impact of intra-band beam variations been quantified for AtLAST? What strategies are planned to mitigate this effect?

 8. Figures 14-15 suggest using an SED-fitting approach, similar to Erler et al. (2018), to separate the relativistic SZ effect from correlated dust contamination. However, this method does not appear to take full advantage of AtLAST's high angular resolution. In contrast, multi-scale, map-based component separation techniques would likely be more effective at distinguishing relativistic SZ corrections from the dust emission of star-forming galaxies in clusters, which occupy much smaller angular scales than the cluster itself. The authors should emphasize how AtLAST's high resolution could enhance this separation, if applicable.

**Minor comments:**

 1. The paper includes a large number of references, but adding explicit definitions and equations for key quantities (e.g., tSZ, rtSZ, kSZ, non-thermal SZ) would improve clarity and self-containment.

 2. In Section 3.2, when discussing current constraints on the relativistic SZ effect, the authors could reference the recent stacking analysis of Planck data by Remazeilles & Chluba (MNRAS, 2025), which used a blind map-based component separation approach.

 3. The right panel's label in Figure 7 is unclear. Is this ACT data alone, or ACT+Planck combined? The caption and body text seem inconsistent and should be clarified.

 4. In Section 4.1, the statement "At the same time, we would like to consider a minimum setup in order not to result in an oversampling of the target spectral range" is unclear. Could the authors clarify? Intuitively, increasing the number of frequency channels should improve foreground cleaning and component separation. Is there a trade-off between the number of channels and instrument sensitivity that limits the spectral sampling to nine channels?

 5. What is "active, closed loop metrology"? A short description/definition could be helpful.

 6. Figure 4 (page 8) is referenced much later in the text (page 15), whereas other figures (e.g., Figure 5) appear earlier. The figures could be reordered to improve the logical flow.

**Typos:**

 - Figure 1 caption: "(ICM) of the" -> "(ICM), and of the"?

 - Section 4.1: "we decide do not include" -> "we decide to not include" or "we decide to exclude"

 - Section 5.2.4: "meta-lrich" -> “metal-rich”

 - Section 6: "mapping the SZ signal from at low or intermediate redshift astrophysical sources" -> Needs rewording (e.g., "mapping the SZ signal from astrophysical sources at low or intermediate redshift")

Is the case presented with sufficient detail to be useful for teaching or other practitioners?

Yes

Is the work clearly and accurately presented and does it cite the current literature?

Yes

If applicable, is the statistical analysis and its interpretation appropriate?

Yes

Are all the source data underlying the results available to ensure full reproducibility?

Not applicable

Are the conclusions drawn adequately supported by the results?

Partly

Is the background of the case’s history and progression described in sufficient detail?

Yes

Reviewer Expertise:

Cosmology, CMB, foregrounds, component separation

I confirm that I have read this submission and believe that I have an appropriate level of expertise to confirm that it is of an acceptable scientific standard, however I have significant reservations, as outlined above.

## References

[1] : Evidence for relativistic Sunyaev–Zeldovich effect inPlanck CMB maps with an average electron-gas temperature ofT e ≃ 5 keV. *Monthly Notices of the Royal Astronomical Society* .2025;538(3) : 10.1093/mnras/staf384 1576-1586 10.1093/mnras/staf384

# Response

**Published:** 2025-05-17

**Authors:** Luca Di Mascolo

**Affiliations:** Kapteyn Astronomical Institute, Rijksuniversiteit Groningen, Groningen, Groningen, The Netherlands

We thank the reviewer for the valuable and detailed report. We include the detailed responses to the individual comments below.

***referee:***

*1. While the manuscript discusses the limitations of current and future submillimeter facilities, a more quantitative comparison with upcoming telescopes such as FYST, CMB-S4, and CMB-HD would strengthen the argument. A graphical representation comparing AtLAST's expected frequency coverage, sensitivity, resolution, and field of view with these facilities would help clarify its unique capabilities and how it complements or surpass other projects.*

***authors: *** Following the referee’s comment, we included a new figure (Fig. 10) for comparing the proposed spectral coverage of AtLAST with past, current, and future (sub)mm facilities. Plots showing AtLAST’s performances in terms of angular resolution and field of view in comparison to other facilities are already provided in past AtLAST works (see, for instance, Klaassen+2020). Booth+2024 also report a comparison of the number counts of detectors for AtLAST and other facilities. As such, we prefer not to include these plots in our manuscript and refer to the aforementioned papers.

***referee: *** 2. Section 3.3 emphasizes the potential of AtLAST for kinetic SZ (kSZ) studies. However, given the inherent spectral degeneracy between the kSZ effect and CMB temperature anisotropies, how does AtLAST intend to disentangle these signals within the ICM? I would expect AtLAST's high spatial resolution to play a crucial role in overcoming this degeneracy, but this advantage is not emphasized in the discussion of the kSZ science case. ***authors: *** This is indeed the case, as we plan to integrate recent advances in statistical data processing in our analysis pipelines. One example is the application of wavelet phase harmonics for performing a data-driven statistical component separation (as tested, for instance, on Herschel measurements for disentangling the CIB from large-scale Galactic dust emission; https://ui.adsabs.harvard.edu/abs/2024A%26A...681A...1A/abstract). We included a brief statement in Sec. 3.3.2 (after splitting Sec. 3.3 in two subsections for the sake of readability).

***referee: *** 3. In Figure 3, the Compton-y profiles evolve with redshift. The reason for this evolution should be explicitly discussed, as the thermal SZ effect is generally considered redshift-independent. How much of this evolution is driven by intrinsic astrophysical effects (e.g., AGN feedback, accretion, non-thermal pressure) versus observational biases (e.g., resolution limits, selection effects)?

***authors: *** The referee is right in underlining the redshift independence of the SZ surface brightness. This is true when considering a system with a given Compton y. However, Fig. 3 shows the evolution of the pressure profiles at fixed mass, considering for this the definition with respect to the critical density at a given redshift (i.e., M500). Since the critical density evolves with cosmic time (redshift), systems with the same M500 but different redshift will exhibit SZ signals with different amplitudes. At the same time, the overall radial extent of the SZ profiles depends on the specific angular diameter distance at the corresponding redshift. 

 Since this could cause some confusion, we included a short statement for explaining the redshift-dependence of the SZ profiles in the figure.

***referee: *** 4. Despite AtLAST's high instrumental sensitivity, it remains unclear whether the residual foreground contamination can be reduced below the thermal SZ confusion noise or if a 'foreground floor' will persist, preventing accurate separation of faint SZ signals(y = 10^-7) in the WHIM or CGM.

 In particular, Figure 10 shows very low residual foreground contamination from the ILC method compared to the tSZ power spectrum across multipoles. However, how do these residuals compare to the tSZ confusion noise and other SZ-related signals, such as kSZ, relativistic SZ corrections, or non-thermal SZ, which AtLAST also aims to extract? If possible, over plotting these additional spectra in Figure 10 would provide a clearer understanding of their relative importance.

***authors: *** The residuals in the Compton y panel of Fig. 11 (previously Fig. 10) results from the application of ILC method on a mock sky realization comprising CMB, infrared and radio backgrounds, as well as kSZ components (we refer to the updated Sect. 4.3.1 for details). As such, the residuals themselves include any residual power associated with CMB and kSZ signals. We however note that, for simplicity, we did not include any relativistic SZ correction. We included a note about this assumption in Sect. 4.3.1.

 To further support the discussion and following the suggestion by another referee, we included a second panel showing the kSZ residuals, along with their comparison with the primary CMB spectrum.

 Finally, we note that the tSZ signal in the left panel of Fig. 11 denotes the power spectrum of the tSZ confusion noise itself. A detailed analysis of the impact of tSZ confusion noise on (sub)mm survey experiments and the impact of the removal of the most massive haloes on the detection capabilities of low-mass clusters was already discussed in Raghunathan+2022, to which we refer for further details. Given the relevance, we included a more explicit reference to the paper.

***referee: *** 5. The low residual foreground levels in Figure 10 suggest that the ILC method is highly effective. However, it is unclear whether this is primarily due to the availability of nine sensitive frequency channels by AtLAST or if it results from simplified assumptions in the foreground simulations. Providing more details on the sky simulations used in Section 4.2 and Figure 10 would help clarify this.

***authors: *** Compared to the previous version of the paper, we significantly expanded the description of the simulation setup used for inferring the ILC residuals. More details can be found in the dedicated Sect. 4.3.1.

***referee: *** Are extragalactic foregrounds (e.g., CIB and radio sources) spatially correlated with the thermal SZ signal in these simulations?

***authors: *** The model currently adopted in our analysis does not encode any correlation between the tSZ effect and the signal from dusty and radio sources. As tested by Raghunathan+2024 in the case of CMB-S4, though, such a term is expected to introduce only a minor bias on the measured SZ effect (<0.2sigma), with the most extreme (and unrealistic) case reaching an upper limit of a 1sigma bias with respect to the true SZ signal. Modelling such an effect in the case of AtLAST-like observations will require a major effort for extending current sky models down to arcsecond scales, along for developing dedicated tools that could allow for a proper spatio-spectral component separation. As such, we decided to clarify that we are not including a correlation term (Sect. 4.3.1), but to extend the discussion only by adding the reference to the paper by Raghunathan+2024 and by briefly mentioning the results reported in that work.

***referee: *** Are the SEDs of extragalactic foregrounds redshift-dependent?

***authors: *** The extragalactic foreground/background components are based on past measurements by the South Pole Telescope (George+2015). As such, any redshift evolution of the source SED is included in the adopted model, as it encodse the inherent properties of the observed population of extragalactic sources. 

***referee: *** 6. The terminology “kinetic SZ spectral distortion” in Section 3.3 is misleading, as the kSZ effect does not introduce a spectral distortion but instead follows the same spectral shape as the CMB. Similarly, the middle panel of Figure 4 labels the "Kinetic SZ Compton y parameter", which is confusing because the kSZ effect is not characterized by a y-distortion. Could the authors clarify whether there is a unit conversion applied to represent kSZ in this way?

***authors: *** We changed “spectral distortion” with “spectral signature”.

 We further updated the central panel of Fig.4 to refer to yksz as “pseudo-Compton y”, consistently with the new definition introduced in Sect. 2.2. This is a common definition in various observational studies of the kSZ found in the literature. 

***referee: *** 7. While beam chromaticity across different frequency bands can be mitigated through deconvolution and reconvolution, intra-band beam variations could introduce spectral distortions and biases in SZ reconstruction, particularly for relativistic SZ and diffuse WHIM studies. Has the impact of intra-band beam variations been quantified for AtLAST? What strategies are planned to mitigate this effect?

***authors: *** We agree with the referee that the impact of beam chromaticity on the effective resolutions might be severe in the case of high-sensitivity broad-band measurements of the SZ effects. However, as demonstrated by Giardiello+2025 (https://ui.adsabs.harvard.edu/abs/2025PhRvD.111d3502G/abstract) in the context of CMB power spectrum reconstruction, the effect of in-band beam variations can be mitigated by means of the integration of a corresponding model component in the data analysis stage. For AtLAST, we aim at exploring similar solutions (along with applications of component separation techniques to the extreme spatial dynamic range of AtLAST) in more detailed and focused follow-up works. Still, given the relevance of beam chromaticity, we decided to include a reference to the aforementioned work in Sect. 4.2 of our manuscript.

***referee: *** 8. Figures 14-15 suggest using an SED-fitting approach, similar to Erler et al. (2018), to separate the relativistic SZ effect from correlated dust contamination. However, this method does not appear to take full advantage of AtLAST's high angular resolution. In contrast, multi-scale, map-based component separation techniques would likely be more effective at distinguishing relativistic SZ corrections from the dust emission of star-forming galaxies in clusters, which occupy much smaller angular scales than the cluster itself. The authors should emphasize how AtLAST's high resolution could enhance this separation, if applicable.

***authors: *** The referee is correct in highlighting the role of AtLAST’s high angular resolution in enhancing the separation of compact sources. In fact, this was already noted in Sect. 4.4.2 and the simulation is specifically aimed at testing the case for diffuse contamination for which it is not possible to take advantage of scale separation. We however noted that it would been worth including a short mention to such a possibility also earlier in the text, and we added a short closing statement in Sect. 4.2.

***referee: *** 1. The paper includes a large number of references, but adding explicit definitions and equations for key quantities (e.g., tSZ, rtSZ, kSZ, non-thermal SZ) would improve clarity and self-containment.

***authors: *** Following the referee’s suggestion, we included a more extensive definition of the thermal, kinetic and relativistic SZ equations in the introduction to the paper. For the sake of readability, we decided to restructure Sect. 2 in subsections, each focusing on one of the SZ flavors discussed in the paper.

***referee: *** 2. In Section 3.2, when discussing current constraints on the relativistic SZ effect, the authors could reference the recent stacking analysis of Planck data by Remazeilles & Chluba (MNRAS, 2025), which used a blind map-based component separation approach.

***authors: *** This was indeed an oversight on our part. We included the reference when mentioning stacking analyses.

***referee: *** 3. The right panel's label in Figure 7 is unclear. Is this ACT data alone, or ACT+Planck combined? The caption and body text seem inconsistent and should be clarified. ***authors: *** It is indeed a combined Planck+ACT Compton y map. We updated the description in the caption and added a reference to Madhavacheril et al. 2020 for details on the generation of the combined ACT+Planck Compton y map.

***referee: *** 4. In Section 4.1, the statement "At the same time, we would like to consider a minimum setup in order not to result in an oversampling of the target spectral range" is unclear. Could the authors clarify? Intuitively, increasing the number of frequency channels should improve foreground cleaning and component separation. Is there a trade-off between the number of channels and instrument sensitivity that limits the spectral sampling to nine channels?

***authors: *** The constraint of finding a minimal spectral configuration is driven mostly by considerations on the future costs. It is indeed true that considering a finer spectral sampling would allow for an improved component separation, but the addition of extra bands would necessarily increase the instrument’s complexity and overall resource requirements. We included a brief mention of this issue in the corresponding paragraph.  

***referee: *** 5. What is "active, closed loop metrology"? A short description/definition could be helpful.

***authors: *** Following a similar suggestion by another reviewer, Sect. 4.3.3 now includes a short description of the “closed-loop” metrology concept (but refer to the dedicated paper by Mroczkowski+2025 for more details).

***referee: *** 6. Figure 4 (page 8) is referenced much later in the text (page 15), whereas other figures (e.g., Figure 5) appear earlier. The figures could be reordered to improve the logical flow.

***authors: *** We improved the overall description of the figures in the main text, and Fig. 4 is now mentioned already in Sect. 3.1.

***referee: *** Typos:

 - Figure 1 caption: "(ICM) of the" -> "(ICM), and of the"?

 - Section 4.1: "we decide do not include" -> "we decide to not include" or "we decide to exclude"

 - Section 5.2.4: "meta-lrich" -> “metal-rich”

 - Section 6: "mapping the SZ signal from at low or intermediate redshift astrophysical sources" -> Needs rewording (e.g., "mapping the SZ signal from astrophysical sources at low or intermediate redshift")

***authors: *** We addressed all the aforementioned typos either directly or as a result of major rewriting of parts of the text and captions.

# Reviewer report: Reviewer response for version 1

**DOI:** 10.21956/openreseurope.18856.r51895

**Published:** 2025-03-27

**Authors:** Elia Stefano Battistelli

**Affiliations:** 1 Sapienza University of Rome, Rome, Italy

**Report for Manuscript: "** **Atacama Large Aperture Submillimeter Telescope (AtLAST) science: Resolving the hot and ionized Universe through the Sunyaev-Zeldovich effect"**

**General Assessment:**

 This paper presents a compelling case for advancing our understanding of the warm/hot ionized gas in the cosmic web through high-resolution, multi-wavelength Sunyaev-Zeldovich (SZ) effect observations. The authors highlight the critical role of this gas phase in the cosmic matter budget and its implications for galaxy evolution and large-scale structure formation. The proposal for a dedicated instrument on the Atacama Large Aperture Submillimeter Telescope (AtLAST) is timely, given the limitations of current facilities. The paper is well-structured and scientifically rigorous. A few clarifications and additions would however strengthen the manuscript. Below, I provide detailed comments and suggestions.

**Strengths:**

1. **Motivation & Scientific Importance:**
  - The paper effectively underscores the significance of the warm/hot gas in the cosmic web as a key component of the baryon budget, connecting galactic to cosmological scales.
  - The emphasis on the SZ effect as a redshift-independent probe is well-justified, as well as the SZ dependence on the electron density particularly important for low-density environments where X-ray emission becomes inefficient. This point should be stressed more clearly in the text.
2. **Technical Feasibility & Innovation:**
  - The proposal for a wide-field, broad-band, multi-chroic instrument on AtLAST addresses critical limitations of existing facilities.
  - The inclusion of theoretical forecasts to define instrumental requirements strengthens the case for technical development.
3. **Clarity & Structure:**
  - The abstract succinctly outlines the scientific goals, methodological approach, and broader implications.
  - The logical flow from the cosmic web’s multi-phase nature to the need for improved SZ observations is compelling.

**Specific Comments and Suggestions for Improvement:**

**General Comments:**

1. **Polarization Capabilities:**
  - It is not clear whether the proposed instrument will include polarization capabilities. Given the importance of polarized signals for probing gas kinematics and magnetic fields, this should be explicitly addressed.
2. **SZ vs. X-ray for Low-Density Environments:**
  - The authors should stress more clearly that the SZ effect is uniquely suited for probing low-density environments (e.g., filaments and voids) where X-ray emission is too faint to detect. This is a key advantage of SZ observations.
3. **Low-Frequency Channel and AME Science:**
  - The low-frequency channel of the proposed instrument could significantly advance the possibility to disentangle diffferent emissions including understanding of Anomalous Microwave Emission (AME). The authors should highlight this opportunity especially considering the angular resolution and the spectral coverage, as it would broaden the scientific impact of the project.
4. **Instrumental Comparisons:**
  - The authors should mention that AtLAST will fill the gap between instruments like the Simons Observatory (SO) and ALMA in terms of angular scale coverage and *uv*-coverage, providing a unique window into the multi-scale nature of the cosmic web.

**Section-Specific Comments:**

**Section 3.2:**

- The authors should cite the seminal work by **Itoh et al. 1998 (**ApJ, 502, 1, 1998) on the SZ effect and explicitly mention the decomposition of the SZ signal in terms of powers of electron temperature ( *T_e*).

**Section 3.3 (Kinetic SZ):**

- The kinetic SZ effect is noted for measuring line-of-sight gas motions. Could SZ polarization be used to probe motions *perpendicular* to the line of sight? Despite being very faint, this would be an interesting point to discuss, as it could open new avenues for studying gas dynamics.

**Section 3.5 (Angular Resolution):**

- The authors should stress more strongly that **high angular resolution** is critical for resolving small-scale structures (e.g., clumps in filaments, CGM around galaxies) and for minimizing confusion with other signals (e.g., dusty galaxies).

**Section 3.6 (Single-Dish Facilities):**

- The statement that other single-dish facilities (e.g., Nobeyama, GBT, SRT) are limited to lower frequencies is inaccurate for the GBT and SRT, as high-frequency instruments like MISTRAL are now being commissioned.

**Section 3.7 (AtLAST Resolution):**

- The claim that AtLAST has **8.3× better resolution** than ACT should specify the frequency (e.g., at 150 GHz, it is closer to **10× better**).
- The observation of A401-A399 in 10 hours may hold for sensitivity, but the sky coverage should be verified, as this is crucial for mapping large-scale structures.

**Section 4 (Instrumentation):**

- Among the cited instruments, the authors may want to include other instruments now being commissioned for instance at the SRT.
- The phrase "current state-of-the-art continuum cameras (e.g., transition edge sensor bolometers or kinetic inductance detectors)" is misleading, as these are **detectors**, not cameras. The authors should cite specific instruments (e.g., MUSTANG-2, NIKA2, MISTRAL) for clarity.

**Section 5.2.2 (Angular Resolution):**

- The statement that medium-resolution experiments are "limited to resolutions approximately 8.33× lower than AtLAST" is overly precise. A more rounded figure (e.g., "~8× lower") would be more appropriate.

**Other Comments:**

- The suggestion of a **1200 GHz band** to resolve dust/SZ degeneracies should be reconsidered, as the SZ signal is negligible at this frequency. The authors should clarify or justify this claim.
- **Figure 10 caption:** The parenthesis after "Sehgal et al., 2019" should be closed.

**Overall Recommendation:**

 This paper makes a persuasive argument for advancing SZ observations to probe the thermal history of cosmic structures. The proposed instrument would fill a critical niche in multi-scale astrophysics and has the potential to transform our understanding of the cosmic web. With the suggested revisions, the manuscript will be suitable for publication.

 I would like to congratulate with the authors for their significant effort in producing this comprehensive study. The manuscript demonstrates substantial dedication to advancing our understanding of the cosmic web through innovative SZ observations, and the proposed instrument concept for AtLAST represents a thoughtful contribution to the field. The thorough theoretical analysis and clear presentation of scientific motivations are particularly noteworthy.

Is the case presented with sufficient detail to be useful for teaching or other practitioners?

Yes

Is the work clearly and accurately presented and does it cite the current literature?

Yes

If applicable, is the statistical analysis and its interpretation appropriate?

Yes

Are all the source data underlying the results available to ensure full reproducibility?

Yes

Are the conclusions drawn adequately supported by the results?

Yes

Is the background of the case’s history and progression described in sufficient detail?

Yes

Reviewer Expertise:

CMB, Sunyaev Zeldovich effect, millimetric instrumentation

I confirm that I have read this submission and believe that I have an appropriate level of expertise to confirm that it is of an acceptable scientific standard.

# Reviewer report: Reviewer response for version 1

**DOI:** 10.21956/openreseurope.18856.r44539

**Published:** 2024-11-08

**Authors:** Laura Salvati

**Affiliations:** 1 IAS, Université Paris-Saclay, CNRS, Orsay, France

The paper presents the upcoming AtLAST telescope and the science that can be done through the observation of hot gas in mm wavelength. The authors present the scientific case in details, discussing the different goals and how they can be achieved with AtLAST.

 I believe the paper is worth getting indexed after minor improvements that I am suggesting below.

 General comments: 

 - the Figures should be described and discussed a bit more in the main text. What are we seeing and what should be the take-home-message from the plots?

 -- example: Fig. 1 showing the different components/processes happening in galaxy clusters and how they can be observed. A short discussion is done only on the caption of the figure, but it would help the reader to present the main points also in the main text.

 -- and same general considerations for the other figures.

 - In the initial part of paper there should be a short introduction to the AtLAST experiment: it is true that this paper is part of a series of papers presenting the mission and science in general, most of them referred to in this paper, but I still believe that each paper should be able to “stand alone”, presenting a very short description of the experiment. Most of the information are spread over the full text, but a general introduction could be useful. 

 Section 2

 - I believe few standard equations to introduce the thermal, kinetic SZ would be useful in this introductory section, instead of just proportionalities proposed in the text.

 - I would also suggest to introduce more in details (and with the equation definition) the relativistic contribution, to help better understanding the discussion in section 3.2 (also related to Te estimation) and in section 4.3.

 - When listing references for SZ observations, I believe more recent papers need to be cited, eg. all the newest catalogs from SPT, ACT, Planck, and ALMA works, etc..

 Section 3

 - when introducing Fig. 2 for the first time, it is worth introducing also Table 1, at least to stress even more the frequency coverage.

 Section 3.1

 - Introducing “hydrostatic mass bias”: worth adding a short definition, apart from citing Biffi’s paper.

 3.3 at the end

 - For patchy kSZ and reionisation contribution in general, please look also at works of Adelie Gorce, such as Gorce et al 2020 (Ref - 1), A&A 640 (2020), A90

 Link between section 3 and 4:

 Section 3 lists all different scientific goals of the experiment, stressing the importance of given resolution, depth, field of view etc..to achieve these goals.

 To help highlighting what are the requirements needed from an instrumental point of view to reach the scientific goals, a short summary (eg. bullet-point style or table) could be useful, maybe at the beginning of section 4, before going into details in section 4.1. I believe all the information is there but there’s a risk they get lost in the long text before 4.1.

 Section 4

 - Fig. 9 is not mentioned in the text.

 - AtLAST-like simulations: the authors mention that the analysis is based on Raghunathan et al. 2022: it would be worth putting more details and in particular explain if the steps discussed after (the flat sky realisations, the foreground modelling, etc..) are part of the Raghunathan et al. 2022 (Ref - 2) analysis or they are in addition to that.

 - some concepts are introduce in the section but not explained, for example in section 4.2 the SZ confusion limit and the closed-loop metrology. It is necessary to add a short definition.

 - Figure 12: there is a line for Te = 0 KeV. What does it mean?

Is the case presented with sufficient detail to be useful for teaching or other practitioners?

Partly

Is the work clearly and accurately presented and does it cite the current literature?

Partly

If applicable, is the statistical analysis and its interpretation appropriate?

Yes

Are all the source data underlying the results available to ensure full reproducibility?

Not applicable

Are the conclusions drawn adequately supported by the results?

Yes

Is the background of the case’s history and progression described in sufficient detail?

Yes

Reviewer Expertise:

Cosmology and Astrophysics

I confirm that I have read this submission and believe that I have an appropriate level of expertise to confirm that it is of an acceptable scientific standard, however I have significant reservations, as outlined above.

## References

[1] : Improved constraints on reionisation from CMB observations: A parameterisation of the kSZ effect. *Astronomy & Astrophysics* .2020;640: 10.1051/0004-6361/202038170 10.1051/0004-6361/202038170

[2] : Study of Resilience in Learning Environments During the Covid-19 Pandemic. *Frontiers in Education* .2022;6: 10.3389/feduc.2021.677625 10.3389/feduc.2021.677625

# Response

**Published:** 2025-05-17

**Authors:** Luca Di Mascolo

**Affiliations:** Kapteyn Astronomical Institute, Rijksuniversiteit Groningen, Groningen, Groningen, The Netherlands

We are grateful to the referee for the useful and insightful comments, which helped us address key aspects of our paper. Our responses to the individual comments in the report are provided below.

***referee: **the Figures should be described and discussed a bit more in the main text. What are we seeing and what should be the take-home-message from the plots?*

*example: Fig. 1 showing the different components/processes happening in galaxy clusters and how they can be observed. A short discussion is done only on the caption of the figure, but it would help the reader to present the main points also in the main text.*

***authors: *** For what concerns Fig. 1 specifically, we decided to move its first mention at the beginning of the new SZ section (Sect. 2). This seems a more appropriate paragraph, as it specifically introduces the SZ effect with respect to the possibility of probing different astrophysical processes.

 Still, we prefer to keep the figure (and the caption) self-contained and not to extensively introduce the figure in the main text, as we intended it as a schematic summary of the different topics discussed in the paper. Given this, we extended the caption and provided forward references to the various subsections of Sect. 3 (“Proposed science goals”) to facilitate the connection between the cartoon and the text itself.

***referee: *** and same general considerations for the other figures.

***authors: *** Following the referee’s suggestions, we decided to review all the figure captions and corresponding discussion in the main text to improve the overall description of the key points of each plot. For the figures, we decided to adopt a “self-contained” approach, so that the main message of the figure is reported in the caption (improving the stand-alone readability of each figure). Accordingly, we edited the caption where such a message was missing.

 Regarding the discussions in the main text, we introduced minor edits to most of the paragraphs including a mention to a figure. The more extensive edits are instead listed below:

- Sect. 3.1 now includes a more detailed and explicit reference to Fig. 3
- We noticed that Fig. 4 was cited only in Sect. 4.1, while it was initially intended as a visual reference for the discussions in Sect. 3.1 and Sect. 3. As such, we included a short description of the thermal SZ panel of the figure at the end of Sect. 3.1, and a corresponding discussion of the kinetic SZ panel in Sect. 3.3.
- We introduced a more extended discussion of Fig. 5 in the second to last paragraph of Sect. 3.5.
- Despite already mentioning Fig. 9, we decided to expand upon the previous reference to better highlight the importance of the high-frequency bands for the reduction of the contamination from dusty galaxies to the SZ effect.
- We improved the description of Figure 11 (previously Fig. 10) in Sect. 4.3.1.
- We corrected a typo in the description of Fig. 17 (previously Fig. 16) in the main body, and introduced a minor edit to the corresponding discussion.

***referee: **In the initial part of paper there should be a short introduction to the AtLAST experiment: it is true that this paper is part of a series of papers presenting the mission and science in general, most of them referred to in this paper, but I still believe that each paper should be able to “stand alone”, presenting a very short description of the experiment. Most of the information are spread over the full text, but a general introduction could be useful.*

***A*** **uthors: **The introduction now includes a short summary paragraph for introducing AtLAST. For consistency with the other works in the ORE series, we however decided to limit the discussion to the very key aspects of the AtLAST design, and refer to the readers to the relevant works.

***referee:  *** I believe few standard equations to introduce the thermal, kinetic SZ would be useful in this introductory section, instead of just proportionalities proposed in the text.

***authors: *** Following the referee’s suggestion, we included a more extensive definition of the thermal, kinetic and relativistic SZ equations in the introduction to the paper. For the sake of readability, we decided to restructure Sect. 2 in subsections, each focusing on one of the SZ flavors discussed in the paper.

 r **eferee: **I would also suggest to introduce more in details (and with the equation definition) the relativistic contribution, to help better understanding the discussion in section 3.2 (also related to Te estimation) and in section 4.3.

***authors: *** Consistently with the refactoring of the tSZ and kSZ sections, we extended the relativistic SZ discussion and provided a more thorough description of the impact of relativistic temperatures and non-thermal electrons on the measured SZ signal. 

 r **eferee: **When listing references for SZ observations, I believe more recent papers need to be cited, eg. all the newest catalogs from SPT, ACT, Planck, and ALMA works, etc.

***authors:  *** More up to date references were already included in the relevant section, but we agree that it is worth expanding upon the previous references. Section 2 now includes a longer introduction to past observational SZ studies, along with more up to date references. For the survey works, we included citations to the most recent cluster samples by Planck, SPT, and ACT. For what concerns the resolved SZ observations, including a complete list would be impractical. As such, we limit this to a subset of relevant works specific to the mentioned topics or to the first analysis with data from a given high-resolution facility. 

***referee: *** when introducing Fig. 2 for the first time, it is worth introducing also Table 1, at least to stress even more the frequency coverage.

***authors: *** We included a direct mention to Table 1 in the introductory paragraph of Section 3.

***referee: *** Introducing “hydrostatic mass bias”: worth adding a short definition, apart from citing Biffi’s paper.

***authors: *** Following the suggestion by the referee, we included a definition of the hydrostatic mass bias right after its first mention in Sect. 3.1

***referee: *** For patchy kSZ and reionisation contribution in general, please look also at works of Adelie Gorce, such as Gorce et al 2020 (Ref - 1), A&A 640 (2020), A90

***authors: *** After the referee’s comment, we realised that the reionization-kSZ paragraph was lacking a proper set of references to past works. We updated this.

***referee: *** Section 3 lists all different scientific goals of the experiment, stressing the importance of given resolution, depth, field of view etc..to achieve these goals.

***authors: *** To help highlighting what are the requirements needed from an instrumental point of view to reach the scientific goals, a short summary (eg. bullet-point style or table) could be useful, maybe at the beginning of section 4, before going into details in section 4.1. I believe all the information is there but there’s a risk they get lost in the long text before 4.1.

 We agree that a list of required characteristics will help guiding the overall discussion in Sect. 4. We now moved the previous introductory text to Sect. 4 in a new subsection (now Sect. 4.1) and added a short bullet list description of the key requirements at the beginning of Sect. 4.

***referee:  *** Fig. 9 is not mentioned in the text.

***authors: *** We mention Fig. 9 in the second paragraph of Sect. 4.1 “Optimizing the spectral setup”, as well as in Sect. 5.1 “AtLAST scientific cross-synergies”. As mentioned above, we expanded the associated discussion about dust contamination in Sect. 4.1.

***referee: *** AtLAST-like simulations: the authors mention that the analysis is based on Raghunathan et al. 2022: it would be worth putting more details and in particular explain if the steps discussed after (the flat sky realisations, the foreground modelling, etc..) are part of the Raghunathan et al. 2022 (Ref - 2) analysis or they are in addition to that.

***authors: *** The description of the simulated maps included in the previous section refers to a specific test aimed at simulating targeted observations rather than survey-mode runs. As such, despite sharing some similarities with the approach adopted by Raghunathan 2022, it has been adapted to allow a more general treatment of arcsecond-level components and for obtaining an independent estimate of the estimated sensitivity. We note that the former implies that the existing models for the foreground and background models have had to be extended down to such angular scales, with potential issues associated with unphysical modes introduced by the model extrapolation. In order to clarify this, we thus included a brief description of the method used to extend the results by Raghunathan 2022 to the AtLAST case, as well as separating the two discussions in separate subsections.

***referee: *** some concepts are introduce in the section but not explained, for example in section 4.2 the SZ confusion limit and the closed-loop metrology. It is necessary to add a short definition.

***authors: *** We reworded the paragraph mentioning the SZ confusion limit to make its definition more explicit. As mentioned now in the text, we define as “SZ confusion limit” the confusion floor in any SZ measurements induced by low-mass haloes that fall below the detection threshold of a given experiment, or are associated (either physically or due to projection effects) with more massive structures.

 Regarding the closed-loop metrology system that will be integrated in AtLAST, we believe that including a thorough description of the proposed designs would fall outside the scope of the present paper. All the technical details are reported in Mroczkowski+2025. We thus decided to include only a minimal description of the concept of “closed-loop” control system, and refer to the aforementioned paper for a more extensive description.

***referee: *** Figure 12: there is a line for Te = 0 KeV. What does it mean?

***authors: *** The Te=0keV denotes the classical spectrum of the thermal SZ effect, i.e., without introducing relativistic corrections for the non-null electron temperature. We included a brief explanatory note in the caption of Fig. 13 (previously Fig. 12).

# Reviewer report: Reviewer response for version 1

**DOI:** 10.21956/openreseurope.18856.r43967

**Published:** 2024-09-27

**Authors:** François-Xavier Désert

**Affiliations:** 1 Institut de Planétologie et d'Astrophysique de Grenoble, Univ. Grenoble Alpes, CNRS, IPAG, Grenoble, France

The paper discusses the fantastic scientific capabilities of Atlast (50m, 2deg FOV) with the SZ toolbox. It describes in detail all aspects of the physics of clusters, where Atlast would give a huge gain. This is not like CMB cosmology, where the gain is measured with a few parameters. Here, the vast astrophysics of the large-scale structure of the Universe would see a giant leap in many ways thanks to Atlast.

 The paper is well written and detailed enough to give us a good idea of the effort involved. Many citations are given in order to grasp all the ideas, which are numerous and very rich. This paper is also a step forward in designing the best survey strategy (wide or deep surveys).

 Nevertheless, I think that there are some unsubstantiated claims about this telescope that must at least be acknowledged: the ability to recover large scales thanks to the large field of view, and the ability to recover very small scales at high frequencies. For large scales, we would need some sky noise simulations to show that 1-2 degree scales can be recovered (but I admit this is beyond the scope of this paper). For small scales, this is a technical issue: how to build a telescope with sufficient surface accuracy on a 50m surface and with sufficient pointing accuracy (also beyond this paper).

 The paper can be accepted if the following comments are addressed.

**Major comment: **Sensitivity problem: For point sources, a 50m telescope is fantastic, as we gain something like the square of the diameter. For extended sources, the sensitivity brightness does not depend on the size of the telescope, but if we look at a given angular size, the gain is like the diameter of the telescope. Atlast can compete with Alma for point sources although it loses by a factor of about 3 in collecting area. Atlast can outperform other telescopes because of its superior diameter, but the gain in surface brightness sensitivity goes only like the diameter (so not a huge factor). I would rather emphasise mapping speed and angular resolution as the definitive winning parameters.

 You need a reference for the Atlast sensitivity calculator. Finally, I don't understand the last two columns of table 1. It seems that this is a telescope that is 10-100 times better for point sources than existing telescopes. For example, an rms of 0.600 mJy in one second at 1mm is unheard of!

 If I convert page 17, the one hour sensitivity in *y* per beam of 2E-6, I get less than 1 mJy in one second, which is also a very optimistic sensitivity. To get *y* to 5E-7 you need 16 hours per FOV, so 64000 hours for 4000 deg^2. At 9000 hours per year that is seven years without any overhead!

**Minor comments: **Fig. 2 describe ntSZ(p=4)

 Tab. 1 : Can you give the *y* sensitivity per sqrt(hour), and the mapping speed (I see some indications in page 17 only). The caption is cryptic to me *“but consider the broad-band re-implementation of the AtLAST sensitivity calculator. The specific frequencies of the band edges correspond to the ones that minimizes the output noise RMS level in the corresponding band per given integration time.”? *What is survey noise?

 There is a claim that large scales can be recovered, say up to the scales of the field-of-view of Atlast. Is that claim based on simulations of the sky noise? Does it depend on the frequencies (I would expect higher frequencies are more difficult to deal with)?

 Fig. 3 : use of theta B10 and B2 is not the best illustration for SZ effect as the sensitivity will mostly be at 2-1mm. Also, for a radius in abscissae, shouldn’t we use theta/2 ? Finally, SZ effect is independent of redshift, if the cluster is resolved (which it is with Atlast). There are clearly some inconsistencies in that figure.

 Fig. 4 : kSZ *y* parameter is a misnomer. It is not a tSZ distortion. We should rather use something like b=beta. tau where b is the adimensional distortion parameter (like DeltaT/T), beta=v/c and tau is the cluster line-of-sight opacity.

 Fig. 8: what is shown in the left panel? What is the colour scale on the right panel?

 Fig 10. What is the kSZ power spectrum? Isn’t that a goal to measure it? To be fair, you address the issue qualitatively at the end of 3.3. Can you extend the figure to 20000 in *l *(claimed on page 10)?

 rtSZ, by measuring the cluster temperature (without X-rays) will give a brand-new avenue in cluster physics. But it will provide results only for the hottest clusters (how many are there) which are the most massive ones.

Is the case presented with sufficient detail to be useful for teaching or other practitioners?

Yes

Is the work clearly and accurately presented and does it cite the current literature?

Yes

If applicable, is the statistical analysis and its interpretation appropriate?

Yes

Are all the source data underlying the results available to ensure full reproducibility?

Yes

Are the conclusions drawn adequately supported by the results?

Partly

Is the background of the case’s history and progression described in sufficient detail?

Yes

Reviewer Expertise:

Clusters of galaxies, CMB, ISM

I confirm that I have read this submission and believe that I have an appropriate level of expertise to confirm that it is of an acceptable scientific standard, however I have significant reservations, as outlined above.
